# Dopamine promotes head direction plasticity during orienting movements

**DOI:** 10.1038/s41586-022-05485-4

**Published:** 2022-11-30

**Authors:** Yvette E. Fisher, Michael Marquis, Isabel D’Alessandro, Rachel I. Wilson

**Affiliations:** 1 Department of Neurobiology, Harvard Medical School, Boston, MA USA; 2 Present Address: Department of Molecular and Cellular Biology, University of California Berkeley, Berkeley, CA USA; 3 Present Address: Chan Zuckerberg Biohub, San Francisco, CA USA

**Keywords:** Spatial memory, Navigation

## Abstract

In neural networks that store information in their connection weights, there is a tradeoff between sensitivity and stability^1,2^. Connections must be plastic to incorporate new information, but if they are too plastic, stored information can be corrupted. A potential solution is to allow plasticity only during epochs when task-specific information is rich, on the basis of a ‘when-to-learn’ signal^3^. We reasoned that dopamine provides a when-to-learn signal that allows the brain’s spatial maps to update when new spatial information is available—that is, when an animal is moving. Here we show that the dopamine neurons innervating the *Drosophila* head direction network are specifically active when the fly turns to change its head direction. Moreover, their activity scales with moment-to-moment fluctuations in rotational speed. Pairing dopamine release with a visual cue persistently strengthens the cue’s influence on head direction cells. Conversely, inhibiting these dopamine neurons decreases the influence of the cue. This mechanism should accelerate learning during moments when orienting movements are providing a rich stream of head direction information, allowing learning rates to be low at other times to protect stored information. Our results show how spatial learning in the brain can be compressed into discrete epochs in which high learning rates are matched to high rates of information intake.

## Abstract (web summary)

A study demonstrates that plasticity in the head direction system in *Drosophila* is modulated by dopamine, which increases learning when reorienting movements are bringing in new spatial information.

## Main

In artificial neural networks, learning is generally restricted to specific epochs when the network is presented with a rich source of training data; then, connections are frozen outside these epochs, to prevent the loss of stored information^4^. By contrast, in biological neural networks, learning is often assumed to be continuous, and not restricted to specific epochs^5^. However, during biological reward learning, dopamine neurons are selectively activated by reward prediction errors, and dopamine release promotes reward learning in response to these errors^6^. Thus, dopamine compresses reward learning into specific epochs when task-specific information is rich. However, it is not clear whether a similar when-to-learn signal also governs other forms of learning, such as unsupervised spatial learning.

**Fig. 1:**
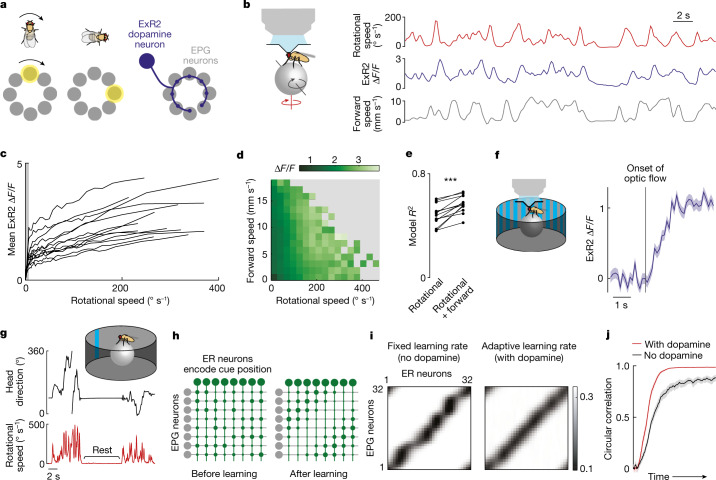
ExR2 dopamine neurons are correlated with rotational speed. **a**, Schematic of the head direction map. **b**, Imaging jGCaMP7f in ExR2 neurons while measuring rotational and forward walking speed. **c**, Mean ExR2 Δ*F*/*F* versus rotational speed (one line per fly, *n* = 13 flies). Grey shading indicates transitions between resting and moving; outside this range, Δ*F*/*F* and rotational speed are linearly related. **d**, Mean ExR2 Δ*F*/*F* binned by rotational and forward speed, aggregated over 13 flies and averaged over time points. Grey bins are empty. **e**, Variance explained (adjusted *R*^2^) for linear regression models that use speed to predict ExR2 activity. Each pair of dots is one fly (*n* = 13). Models were fitted separately for each fly. Rotational speed alone produced a high *R*^2^; adding forward speed produced a small additional increase (***P = 5.3 × 10^−5^, two-sided paired *t*-test). **f**, ExR2 responses to optic flow. A stationary vertical grating begins to rotate, and the onset of optic flow drives a sustained increase in ExR2 activity (mean ± s.e.m. across flies; Δ*F*/*F* is significantly different from zero with *P* = 0.0012, two-sided one-sample *t*-test, *n* = 13 flies). Here we analysed only trials when the fly was standing still. **g**, Example data used as model input. Flies walked in a virtual environment with a visual head direction cue. **h**, Schematic ER-to-EPG connectivity. Adjacent ER neurons in the schematic have adjacent receptive fields in azimuthal space. Connection weights are denoted by circle sizes. Weights are initialized randomly, and then evolve through Hebbian plasticity. **i**, Weights from a typical model run. **j**, Mean circular correlation between the population vector average of ER output weights and EPG input weights; mean (*n* = 117 simulations trained on shuffled data) ± 95% confidence interval. At the end of the simulation, the correlation is higher with the adaptive learning rate (*P* = 6.2 × 10^−21^, two-sided Wilcoxon sign rank test).

**Extended Data Fig. 1:**
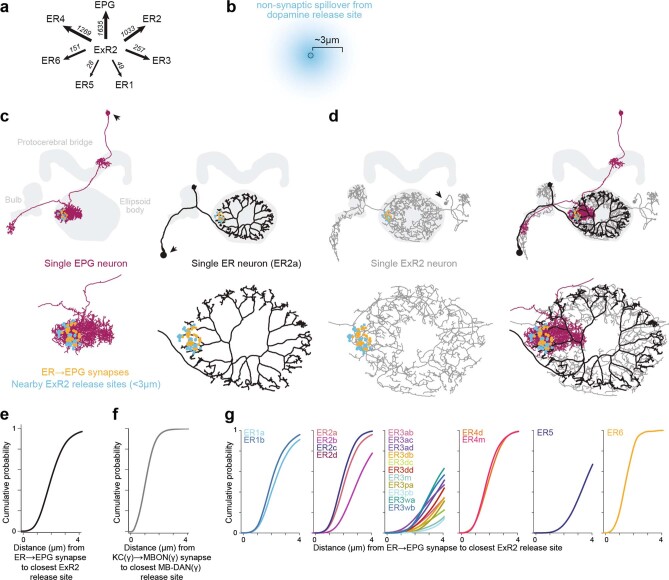
Proximity of ExR2 dopamine release sites to ER→EPG synapses. All data in this figure originate from the ‘hemibrain’ large-scale serial section electron microscopy dataset^57^. a) Total number of synaptic contacts from ExR2 neurons onto EPG neurons and ER neurons. b) Schematic of dopamine spillover. Work in the *Drosophila* mushroom body has suggested that dopamine can act on synapses up to ~3 µm away from its sites of release^74^. This motivated us to count ER→EPG synapses <3 µm from an ExR2 release site; here we consider all ExR2 release sites, regardless of the postsynaptic cell. This analysis assumes that plasticity at ER→EPG synapses is regulated by dopamine receptors residing at or near the synapse, rather than dopamine receptors elsewhere in ER or EPG neurons. c) Skeleton of one EPG neuron and one ER neuron (ER2a subtype). Gray regions show neuropil boundaries. ER→EPG synapses between these two neurons are shown in gold. Bottom images show the ellipsoid body at an enlarged scale. Nearby ExR2 release sites are in blue (<3 µm from a gold site). Arrows denote cell body locations. d) Skeleton of one ExR2 neuron. There are four ExR2 neurons in total (two in each hemisphere), and the blue release sites come from all four of these neurons. At right is an overlay of the example cells shown in (c). Arrow denotes ExR2 cell body location. e) Cumulative probability histogram of Euclidean distances from all visual ER→EPG synapses to the closest ExR2 release site. We define “visual ER neurons” as subtypes ER2a, ER2b, ER2c, ER2d and ER4d^25,32,82^. f) Cumulative probability histogram of distances in the mushroom body γ lobe from Kenyon cell → mushroom body output neuron synapses (KC→MBON synapses) to the closest mushroom body dopamine neuron (MB-DAN) release sites. Note that the proximity of dopamine release sites to KC→MBON synapses is similar to the proximity of release sites to ER→EPG synapses. See also ref. ^74^. g) Cumulative probability histogram of the distance from each ER→EPG synapse to its closest ExR2 release site, plotted separately for each ER subtype.

During spatial learning, task-relevant information comes from movement through space, which could provide a useful when-to-learn signal. Indeed, some dopamine neurons are time-locked to movement^7–14^, and even to specific kinematic variables such as forward acceleration of the body, or rotational velocity of the head^15–18^. Movement-locked activity has also been noted in certain dopamine neurons in the *Drosophila* *melanogaster* brain^19–23^. These include ExR2 neurons^22^, which provide dopaminergic input^24–26^ to head direction cells^27^, also known as EPG neurons (Fig. 1a and Extended Data Fig. 1). EPG neurons can rapidly learn new visual cue configurations when the fly enters a new environment, probably through Hebbian plasticity at the synapses from visual ER neurons onto EPG neurons^28,29^; however, this type of spatial learning should be allowed only when the fly is actively changing its head direction, to avoid creating biases in the head direction map when the fly’s gaze is stationary—in essence, to avoid ‘over-learning’ any particular snapshot of the visual scene^29^. We wondered whether ExR2 neurons are selectively active when the fly is changing its head direction and, if so, whether these dopamine neurons promote associations between visual cues and head directions.

## Dopamine neurons that track rotations

**Extended Data Fig. 2:**
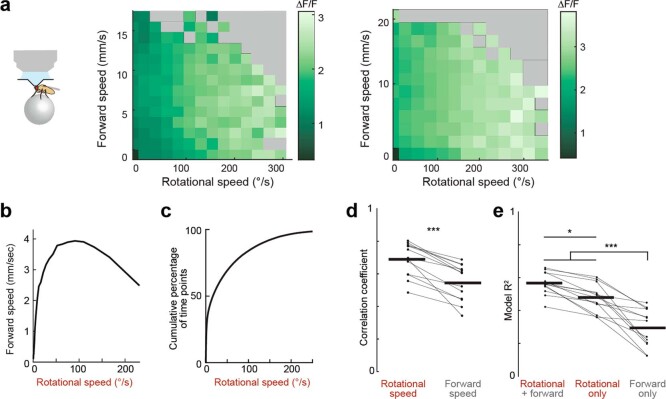
Rotational speed tuning in ExR2 dopamine neurons. a) Mean ExR2 ΔF/F versus rotational and forward speed for two example flies. Data are binned by speed and averaged over time points. Gray bins are empty. Figure 1d shows the average over all time points for all flies. b) Mean forward speed versus rotational speed for the combined behavioral data of all 13 flies in Fig. 1c–f. Data are binned by rotational speed and then averaged across time samples. Note that rotational and forward speed are positively correlated for rotational speeds <100 °/s. c) Cumulative histogram of rotational speed data from this data set. Note that rotational speed <100 °/s for the large majority of time points; thus, rotational and forward speed are generally positively correlated. d) Pearson’s correlation coefficient between ExR2 ΔF/F and rotational or forward speed. Each set of connected dots represents an individual fly (n = 13); horizontal lines are mean values. ExR2 activity is more correlated with rotational speed than with forward speed (p = 0.0002, two-sided Wilcoxon signed rank test). The positive correlation with forward speed is expectable, based on the fact that rotational and forward speed are themselves correlated. e) Comparison of the explanatory power (adjusted R^2^ values) of linear regression models that aim to predict ExR2 ΔF/F using rotational and forward speed, rotational speed only, and forward speed only. In Wilkinson notation, the model formula is ΔF/F ~ speed_rotational_ + speed_forward_. Each set of connected dots represents an individual fly; horizontal lines are means (n = 13 flies). Models were fit separately for each fly. Models that used only forward speed as a predictor variable performed significantly worse than those that used rotational speed (p = 3.1 × 10^−5^), and models that used both forward and rotational speed performed better than those using rotational speed alone (p = 0.04, one-way repeated measures ANOVA with Tukey post hoc test). However, the incremental benefit of adding forward speed was small, implying that forward speed is mainly predictive of ExR2 activity simply because it is predictive of rotational speed.

**Extended Data Fig. 3:**
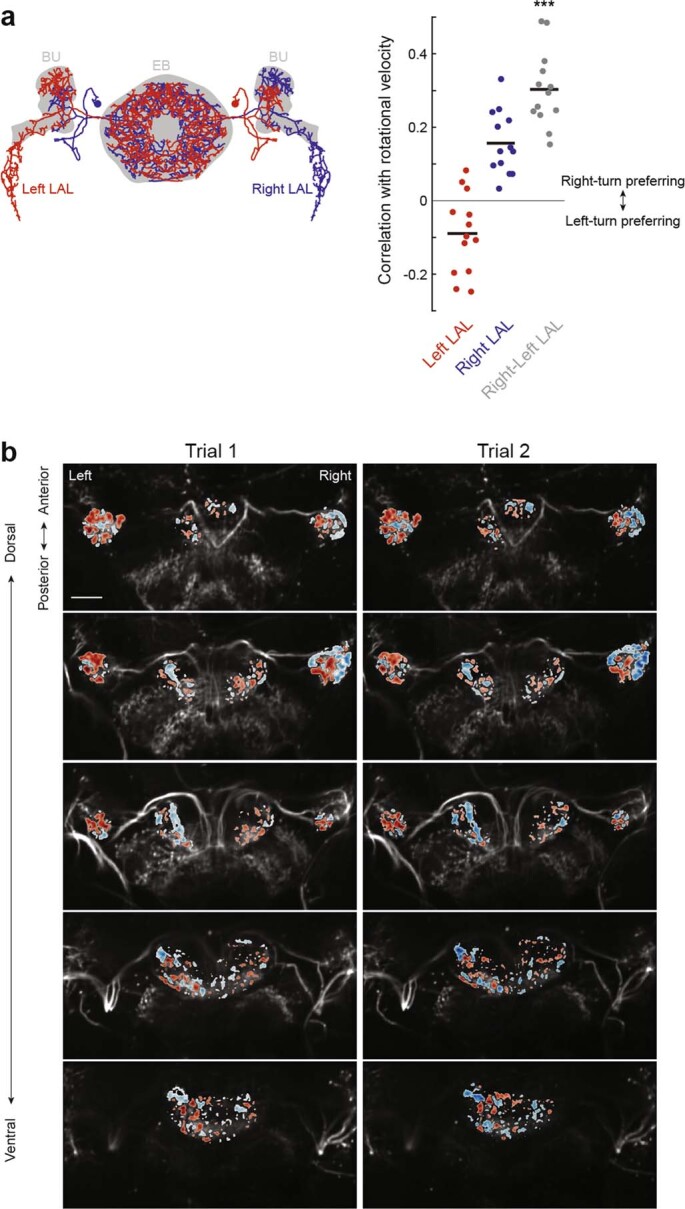
Lateralized ExR2 responses to rotational movements. a) *Left*: Skeleton of one ExR2 neuron (from the hemibrain dataset) overlaid with a mirrored version of the same skeleton. Note that the LAL dendritic arbor is mainly ipsilateral (not bilateral); therefore, we analyzed ExR2 GCaMP7f fluorescence in the LAL to compare the velocity tuning of the left and right copies of ExR2. There are four ExR2 neurons in total (2 cell bodies in each hemisphere). *Right*: Pearson’s correlation between ExR2 ΔF/F in the LAL and the fly’s rotational velocity. Rotational velocity is defined as positive for rightward turning and negative for leftward turning (as distinct from rotational speed, i.e. the absolute value of rotational velocity, which is strictly non-negative). This analysis shows that ExR2 signals are correlated with rotational velocity in the ipsilateral direction, and the right-left difference in ExR2 activity is significantly correlated with the fly’s rotational velocity (correlation significantly different from zero, p = 1.8 × 10^−7^, two-sided one-sample t-test). Each fly contributes one data point per condition (n = 13 flies). b) Pearson’s correlation with rotational velocity overlaid on raw fluorescence images acquired at 5 different horizontal planes through the same brain, for two different 5-minute trials with a 5-minute period between them. Scale bar is 20 μm. Correlation coefficients are represented by a blue-to-red colormap, with R > 0 in blue and R < 0 in red, with stronger absolute correlations having more saturated color values. Correlation values are only shown for the pixels with the strongest correlations (top 15% of absolute correlation values). Note that many pixels have consistent tuning across the two trials. Pixels with positive and negative correlations are likely to arise from the right and left copies of ExR2, respectively. This analysis demonstrates that the direction-selectivity we see in the LAL arbors is preserved in the EB and BU arbors of these cells. If dopamine release from each ExR2 neuron is proportional to the fly’s ipsilateral rotational velocity, then total (summed) dopamine release in the EB should be proportional to the fly’s rotational speed.

To investigate this hypothesis, we carried out two-photon calcium imaging of ExR2 axons as flies walked on a spherical treadmill in darkness (Fig. 1b), using a selective transgenic line to drive expression of jGCaMP7f in these cells. There are four ExR2 neurons per brain, and we imaged all their axons simultaneously. We found that ExR2 neurons are most active when a fly turns, thereby changing its fictive head direction (Fig. 1c). On a moment-to-moment basis, there is a nearly linear relationship between ExR2 activity and the fly’s rotational speed (Fig. 1d). Linear regression shows that rotational speed explains much of the variance in ExR2 activity (Fig. 1e and Extended Data Fig. 2). These rotational speed signals in ExR2 neurons probably reflect internal copies of motor commands and/or proprioceptive feedback from the legs. These signals may arise from the synaptic inputs to ExR2 neurons in the lateral accessory lobe, a brain region that issues descending steering commands^25^. Specifically, rightward steering commands are driven by activity in the right lateral accessory lobe, and vice versa^22,30^. We found the same lateralization of activity in ExR2, and the summed output of all four ExR2 neurons scales with rotational speed in either direction (Extended Data Fig. 3).

**Extended Data Fig. 4:**
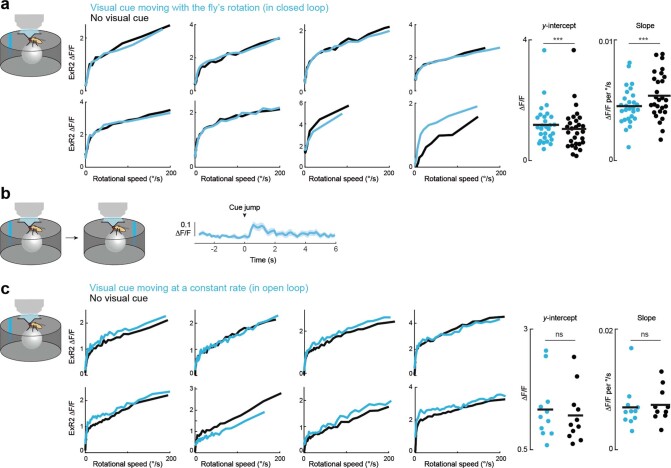
Small ExR2 responses to the movement of a visual object. a) We placed flies in a virtual reality environment with a visual cue which rotated around the fly in closed loop with its rotational velocity on the spherical treadmill. In an environment with this type of visual cue, EPG neurons track the fly’s head direction more accurately than they do in darkness^27^; here we ask whether this type of cue affects ExR2 activity also. *Left*: mean ExR2 ΔF/F versus the fly’s rotational speed for 8 example flies. Right: slopes and *y*-intercept of lines fit to the data for each fly in the linear portion of each curve (rotational speeds >30°/sec), with one data point per fly. In the epochs with the visual cue, slopes were significantly smaller and intercepts were larger, compared to epochs of darkness in the same experiments (slope: p = 6.3 × 10^−5^, intercept: p = 0.0004, two-sided paired t-tests with Bonferroni correction, n = 29 flies). This indicates that the visual cue boosts ExR2 activity for low rotational speeds; however, the magnitude of this effect is very small. These results are consistent with the fact that ExR2 neurons respond to optic flow (Fig. 1f), and the movements of the cue produce a small amount of optic flow. b) We also tested the effect of jumping the visual cue by 90° during these closed-loop epochs; we found that this produced a very small and transient ExR2 response, which is likely due to the small transient increase in optic flow that the cue jump produces (compare with Fig. 1f). Shown here is the average response from a typical example fly (mean of 11 trials ± SEM). To assess the effect of the cue jump, we analyzed only those trials where the fly happened to be standing immobile for several seconds before and after the jump, in order to avoid confounds associated with jump-induced behaviors. c) Here we compare epochs of walking in darkness with epochs of open-loop cue rotation at constant velocity (with the same cue velocity used in Figs. 2, 3, and 4, i.e. ~18°/s). *Left*: mean ExR2 ΔF/F versus the fly’s rotational speed for 8 example flies. Data are binned by rotational speed and averaged across time points. *Right*: slope and *y*-intercept of lines fit to the data for each fly in the linear portion of the curves (rotational speeds >30°/s). Visual cue rotation at constant velocity had no significant effect on the relationship between ExR2 activity and the fly’s rotational speed (slope: p = 0.87, *y*-intercept: p = 0.15, two-sided paired t-tests with Bonferroni correction, n = 11 flies). However, this data set shows a small trend in the same direction as what we observed in the closed-loop case; because there are fewer replicates here, there is less statistical power. The main result of this experiment is simply that we find no evidence that open-loop cue movement produces any *larger* response than closed-loop cue movement does. The flies in this panel are the same as those shown in Fig. 1c–f.

Rotational speed can also be signalled by rotational optic flow^31^, and indeed we found that ExR2 neurons are robustly activated by widefield rotational optic flow, even when flies are standing still (Fig. 1f). Thus, ExR2 neurons combine multiple sources of information (both non-visual and visual) to estimate rotational speed. ExR2 neurons show only a small response to the movement of an object within the visual scene, consistent with the fact that a moving object generates a relatively small amount of optic flow, compared with a widefield rotation of the entire scene (Extended Data Fig. 4).

## A model with an adaptive learning rate

We then adapted a published computational model^29^ to explore the consequence of ExR2 activity for spatial learning. The input to the model was a temporal sequence of visual cue positions and associated rotational velocities. We took this sequence directly from behavioural experiments in which we allowed head-fixed walking flies to walk in a virtual reality environment with a visual cue; when the fly attempted to turn, this caused the visual cue to rotate around its head in the expected direction (Fig. 1g). In the model, this recorded sequence of virtual gaze directions was used to drive the visual ER neurons that project to head direction cells^32^. Model ER neuron receptive fields evenly tiled the space of head directions, so that activity moved across the ER array as the fly turned. In the model, each ER axon connected to all head direction cells (EPG neurons), and ER-to-EPG synaptic weights were plastic. To ensure a stable bump of EPG activity (attractor dynamics), model EPG neurons were linked by reciprocal short-range excitation and global inhibition^33^. At the outset of each simulation, ER-to-EPG weights were initialized randomly and then allowed to evolve over time, following a Hebbian bidirectional learning rule, until they reached a stable equilibrium (Fig. 1h,i). Consistent with the findings of previous work^29^, our observations showed that learning in this network produces an irregular pattern of ER-to-EPG weights (Fig. 1i). This irregularity arises from the fact that the fly often orients in a fixed direction for long periods, and so over-learns the cue location that is associated with that direction.

**Extended Data Fig. 5:**
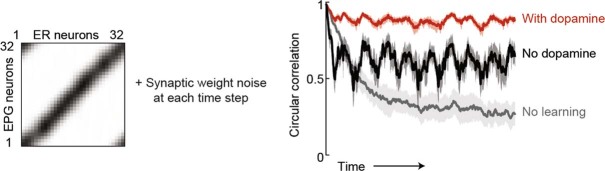
Model illustrating the effect of synaptic plasticity in combating synaptic weight noise. Here we began with weights from the end of a typical model run with an adaptive learning rate (“dopamine”). As before, the input to the model was a temporal sequence of visual cue positions and associated rotational velocities, taken from our behavior data (as shown in Fig. 1g). At each time step, we then made small random changes to individual synaptic weights. These random changes could represent (for example) fluctuations in the numbers of presynaptic calcium channels or postsynaptic neurotransmitter receptors. If the Hebbian learning rule continues to operate, with the adaptive learning rate (“dopamine”), then as the fly continues to walk and sample the visual environment, the effects of these small random changes are erased, and the synaptic weight pattern is preserved. But without dopamine, the synaptic weight pattern degrades, because the fly often orients in a fixed direction for long periods, and so over-learns the retinotopic cue location that is associated with that direction. Finally, if learning is turned off, synaptic weights degrade very rapidly, because the random synaptic weight changes accumulate without any correction. The plot shows the mean circular correlation between the population vector average of ER output weights and EPG input weights; mean (n = 16 simulations trained on shuffled data) ± 95% confidence interval. At the end of the simulation, all pairwise comparisons between conditions are significantly different (normal learning vs. fixed rate learning: p = 0.0004; normal learning vs. no learning: p = 0.0004; fixed rate learning vs. no learning: p = 0.0006; two-sided Wilcoxon sign rank tests with Bonferroni correction).

Then we added dopamine neurons to the model so that the weight change at each time step was scaled by dopamine neuron activity, which was taken as proportional to the fly’s rotational speed, as per our ExR2 imaging data. This adaptive learning rate produces a more regular pattern of synaptic weights (Fig. 1i,j). This occurs because dopamine neurons are active only when the fly is actively changing its head direction, and so if dopamine regulates the learning rate, learning can occur only during the epochs in which there is a rapid sampling of gaze directions. Finally, even after the network develops a regular pattern of synaptic weights, Hebbian plasticity and dopamine neurons are still useful, because they combat the ongoing effect of synaptic weight noise (Extended Data Fig. 5). These model results support a previous theoretical framework that assumed a slightly different formulation of the adaptive learning rate^29^.

## Pairing dopamine with a direction cue

**Fig. 2:**
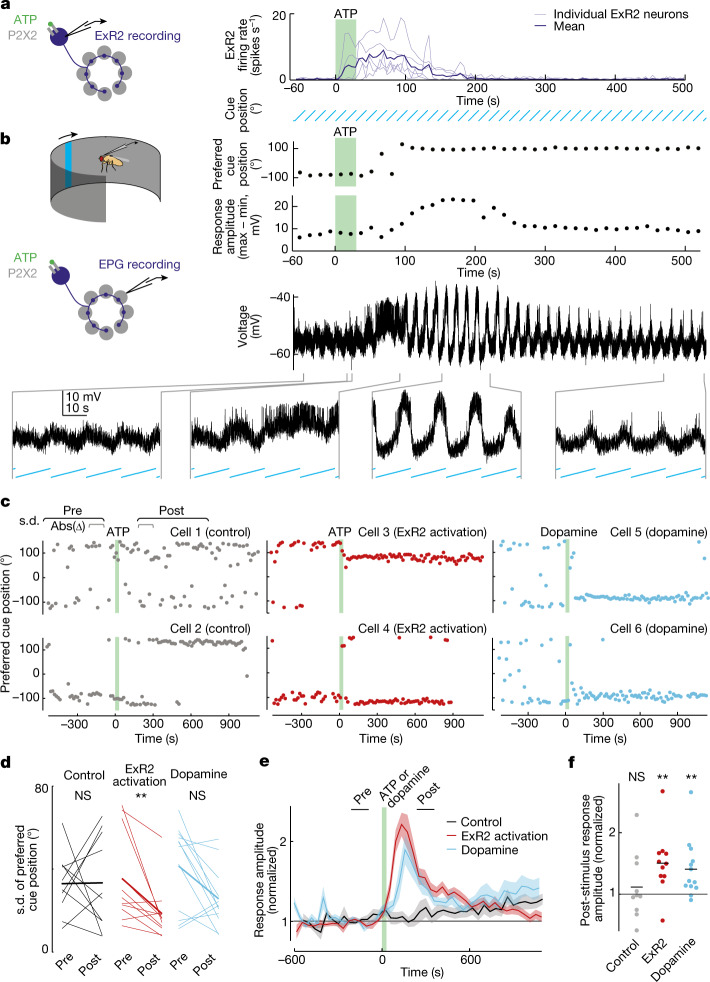
ExR2 dopamine strengthens the association between EPG neurons and a visual cue. **a**, A 30-s pulse of ATP (5 mM) excites ExR2 neurons expressing P2X2 receptors (*n* = 5 cells). The fly is not standing on a spherical treadmill in this figure or in Fig. 3. **b**, An example EPG neuron responding to a rotating cue. For each cue cycle, we measured the neuron’s preferred cue position and its response amplitude (maximum − minimum membrane potential). A position of 0° means the cue is in front of the fly. Extended Data Figure 6a shows another example. **c**, Preferred cue position over time for six EPG neurons. Each point is one stimulus cycle. The green shading shows the pulse of ATP (5 mM) or dopamine (200 µM). In controls (cells 1 and 2), ExR2 neurons did not express P2X2 receptors. With ExR2 activation (cells 3 and 4), the cell’s preferred cue position became more consistent, and it sometimes shifted. Dopamine produced similar changes (cells 5 and 6). s.d., circular standard deviation. **d**, Variability of preferred cue position, before and after ExR2 activation (*n* = 11) or dopamine (*n* = 12) versus ATP treatment in controls in which ExR2 neurons do not express P2X2 (controls, *n* = 10). The fine lines represent individual EPG neurons; the thick lines represent means. The preferred cue position becomes less variable after ExR2 activation (**P = 0.0049). Dopamine produces a similar trend, although falling short of significance (not significant (NS), *P* = 0.052). ATP has no effect in controls (NS, *P* = 0.77, two-sided Wilcoxon sign rank tests). The values are measured over the windows shown in **c**. **e**, Amplitude of the response to the visual cue, normalized to each cell’s baseline, averaged over cells (±s.e.m.); *n* values as in **d**. **f**, The normalized response amplitude increases after ExR2 activation (**P = 0.0068) or dopamine treatment (**P = 0.0024) but not in controls (NS, *P* = 1, two-sided Wilcoxon sign rank test). The dots represent single cells; the lines represent means; *n* values as in **d**.

**Extended Data Fig. 6:**
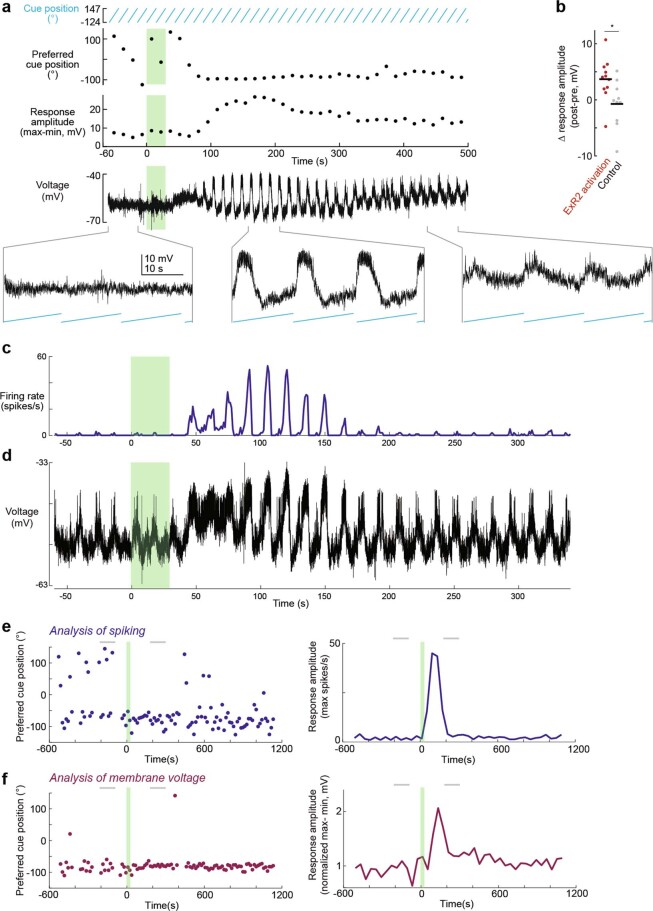
Additional examples of visual response changes and spiking in EPG neurons. a) Example EPG neuron responding to a rotating visual cue, recorded in whole-cell mode. For each stimulus cycle, we measured the neuron’s preferred cue position and its response amplitude (max - min membrane potential). Insets show membrane potential on an expanded timescale. In this EPG neuron, ExR2 activation produced a relatively long-lasting increase in visual cue response amplitude, whereas the increase was more transient in the example EPG neuron in Fig. 2b. b) Across all recorded cells, the change in visual cue response amplitude was significantly higher after ExR2 activation, as compared to control experiments where ATP was applied but ExR2 neurons did not express P2X2 (p = 0.015, two-sample two-sided Wilcoxon rank sum test). Dots are individual cells (same cells as in Fig. 2); horizontal lines are means; n = 11 for ExR2 activation and n = 10 for control. Changes in visual cue response amplitude (post − pre) are measured in the time windows shown in Fig. 2e. c) Example EPG spike rate changes following ExR2 activation during the rotation of a visual cue. In this EPG neuron, spikes are unambiguously identifiable, but that was not true in all recordings, because ExR2 activation causes an acute depolarization and a large (and often persistent) increase in visually-evoked EPG spike rates. Under these conditions, the EPG spike amplitude decreases, making spike identification uncertain in many recordings. d) Membrane voltage response for the same cell during the epoch as shown in (c). e) Analysis of spike rates for the same example cell in (c). Preferred cue position over time (left) and amplitude of the response to the visual cue (bottom). Each point is one stimulus cycle. f) Same but for membrane potential. The response amplitude is normalized to the cell’s baseline period.

**Extended Data Fig. 7:**
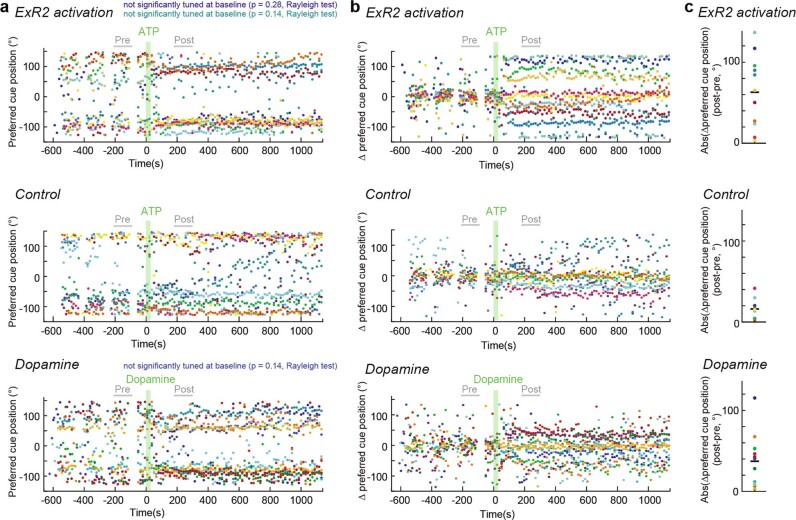
Preferred visual cue positions of individual EPG neurons. a) Preferred visual cue position over time, for all EPG neurons single-cell recordings (Fig. 2b–f). Each color denotes a different cell (n = 11 for ExR2 activation, n = 10 for controls, n = 12 for dopamine). Preferred cue position was measured once per cue rotation cycle. To assess the significance of baseline tuning, we applied the Rayleigh test for non-uniformity to the entire baseline period, using the circ_rtest() function in Matlab^77^; two cells in the ExR2 activation dataset and one cell in the dopamine dataset were not significantly tuned to visual cue position during the baseline period. These cells and the corresponding p-values are annotated above the plot (with p values from Rayleigh tests indicating the probability that the cell is untuned). Note that preferred cue positions tend to be biased toward +90° and −90°, and this bias becomes more prominent following ExR2 activation or dopamine treatment. This bias is probably inherited from visual ER neurons, which overrepresent these lateral positions^32^; as a result, many EPG neurons tend to receive disproportionate inhibition at these locations^28^, and thus they might be expected to receive disproportionate disinhibition at the opposite location (−90° and +90°, respectively). This bias should be less notable when the visual stimulus is reinforcing the internal self-motion inputs to the head direction system (i.e., motor efference and/or proprioceptive inputs^35,36^), because these self-motion inputs presumably do not exhibit any retinotopic bias. Accordingly, a recent study found a strong egocentric bias in butterfly head direction cells during passive viewing of a visual cue, but not when the butterfly’s steering movements were controlling the position of that same visual cue^83^. If the bias arises from the properties of visual ER neurons, then we might expect the bias to increase when the influence of the visual cue increases, and indeed this is what we observe when we pair the visual cue with ExR2 activation or dopamine treatment. b) Same as (a) but expressed as a change from the cell’s baseline average preferred cue position. c) Absolute value of the change in preferred cue position. Dots are single cells, line is mean. Cells without significant baseline tuning are included in this plot but excluded from all statistical analysis of single cell remapping. N values are the same as in (a).

**Extended Data Fig. 8:**
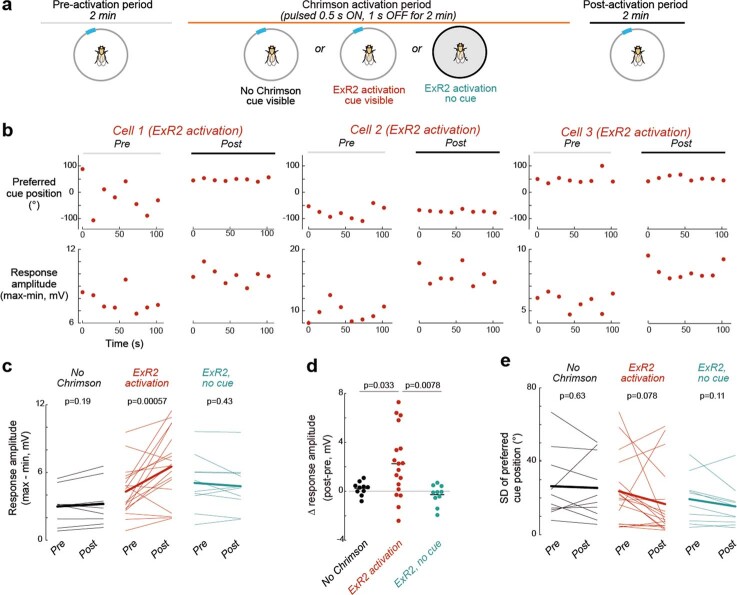
Effects of optogenetic ExR2 activation. a) Schematic of the experimental design. The voltage response of an EPG neuron to a rotating visual cue was obtained in whole cell recording mode, and visual tuning was measured in the pre- and post-activation periods by rotating a visual cue around the fly. During the activation period, orange light was pulsed to activate Chrimson-expressing ExR2 neurons while we continued to rotate the visual cue around the fly. We performed the same protocol in genetic controls where ExR2 neurons did not express Chrimson. In a separate set of controls, we used Chrimson-expressing ExR2 neurons but we turned off the visual cue during ExR2 activation. b) Preferred cue position and response amplitude (max - min membrane potential) for 3 example EPG neurons, pre and post ExR2 activation with Chrimson; all these flies had the visual cue paired with ExR2 activation. Each point is one stimulus cycle. c) Mean response amplitude, pre and post activation. P-values show results of Wilcoxon paired, two-sided rank tests. Results are similar to Fig. 2f (chemogenetic activation of ExR2). (n = 10 for no Chrimson, n = 18 ExR2 activation, n = 10 ExR2 activation with no cue). d) Changes in visual cue response amplitude. Horizontal lines are means. P-values show results of a two-sample two-sided Wilcoxon rank sum tests (n = 10 for no Chrimson, n = 18 ExR2 activation, n = 10 ExR2 activation with no cue). Results are similar to Extended Data Fig. 6b (chemogenetic activation of ExR2), although the absolute change in response amplitude is smaller than with chemogenetic activation. e) Standard deviation of prefered cue position, pre and post activation. P-values show results of Wilcoxon paired, two-sided rank tests (n = 10 for no Chrimson, n = 18 ExR2 activation, n = 10 ExR2 activation with no cue). Compared with Fig. 2d (chemogenetic activation of ExR2), the same trends are visible, although the effect of ExR2 activation falls short of statistical significance. This may be related to the fact that the absolute change in response amplitude was smaller with optogenetic activation than with chemogenetic activation. Overall, for ExR2 activation with Chrimson, 16 of 18 cells were significantly tuned to cue position during the baseline (“pre”) period; of those, 3 changed their preferred cue position after we delivered orange light to activate ExR2 neurons. For the no Chrimson condition, 9 of 10 cells were significantly tuned during the baseline period and 0 of those cells changed tuning after we delivered orange light. For the ExR2 activation with no cue condition, 10 of 10 cells were tuned during the baseline period and 2 showed changes in tuning after we delivered orange light. Tuning during the baseline period was assessed with a Rayleigh test for non-uniformity (at the p > 0.05 criterion for significance). Changes in preferred cue position tuning were assessed using parametric Watson-Williams multi-sample tests with Bonferroni correction.

Next, we experimentally tested the effect of stimulating dopamine release. We reasoned that if dopamine increases the learning rate at ER-to-EPG synapses, then pairing dopamine with a visual cue should increase the cue’s influence on EPG neurons. To stimulate dopamine release, we expressed ATP-gated ion channels (P2X2 receptors) in ExR2 neurons (Fig. 2a). We used ExR2 electrophysiological recordings to verify that a 30-s bath application of ATP produced a transient depolarization and spike rate increase in these neurons (Fig. 2a). Then, in separate experiments, we used electrophysiological recordings to continuously monitor the preferred cue positions of individual EPG neurons as we rotated a visual cue around the fly at a constant velocity (Fig. 2b). We found that ExR2 activation typically caused the cell’s visual tuning to become more consistent, as indicated by a persistent decrease in the cycle-to-cycle jitter of the neuron’s preferred cue position (Fig. 2b–d). In a small subset of cells, there was also an abrupt remapping of the preferred cue position (Fig. 2b,c, Methods and Extended Data Figs. 6 and 7). We saw this visual cue remapping in cells in which the visual cue preferences were already consistent before ExR2 activation (Fig. 1b and Extended Data Fig. 7). These changes generally persisted for as long as we held the recording (10–15 min). At the same time, ExR2 activation also caused a large increase in the amplitude of the EPG neuron’s visual response (Fig. 2b,e,f); this increase typically persisted for several minutes, but it was not as long-lasting as the change in tuning consistency, arguing that the measured change in tuning consistency was not simply a consequence of larger visual responses. In control experiments in which we applied ATP but ExR2 neurons did not express P2X2 receptors, we did not observe any of these effects (Fig. 2c–f). Together, these data indicate that a transient burst of ExR2 dopamine neuron activity can persistently increase the consistency of visual cue responses in head direction neurons, while also causing a remapping of the preferred cue position in a small subset of cells. When we activated ExR2 neurons optogenetically, rather than chemogenetically, we found similar results (Extended Data Fig. 8). Finally, we found that a 30-s bath application of dopamine mimicked some of the effects of stimulating ExR2 neurons (Fig. 2c–f and Extended Data Fig. 7), although its effects were less consistent, possibly because bath application does not match physiological dopamine concentrations or release kinetics.

**Fig. 3:**
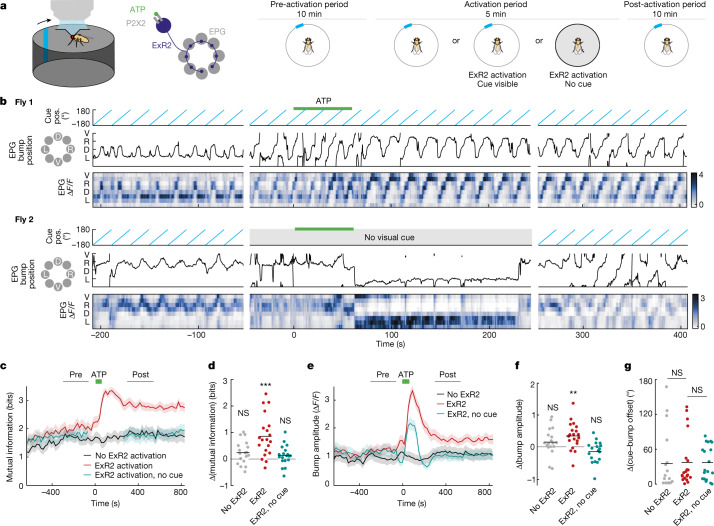
Pairing ExR2 activation with a visual cue increases the cue’s influence on the head direction map. **a**, Imaging jGCaMP7f in EPG neurons while rotating a visual cue around the fly. ATP was delivered during the activation period. In some experiments, ExR2 neurons did not express P2X2 receptors (‘no ExR2 activation’) or ATP was delivered in darkness (‘no cue’). **b**, Example data from two flies. The EPG bump position (pos.) is the angular phase of the bump within the ellipsoid body. In each Δ*F*/*F* heatmap, adjacent rows are adjacent wedges of the circular map in the ellipsoid body. **c**, Mutual information between the cue position and the EPG bump position, estimated for each cue rotation cycle; mean ± s.e.m. across flies (ExR2 activation, *n* = 20; no ExR2 activation, *n* = 18; ExR2 activation with no cue, *n* = 19 in this and all subsequent panels). **d**, Change in the mutual information between the cue position and the bump position (post − pre), measured in the windows shown in **c**. Dots represent flies; lines represent means (no ExR2 activation, NS, *P* = 0.12; ExR2 activation, ***P = 0.00036; ExR2 activation with no cue, NS, *P* = 0.49; two-sided one-sample *t*-tests with Bonferroni correction). **e**, Bump amplitude for each cue rotation cycle (maximum − minimum Δ*F*/*F*); mean ± s.e.m. across flies. When no cue was visible, the bump amplitude was calculated in the equivalent time windows. **f**, Change in bump amplitude (post − pre), measured in the windows shown in **e**. Dots represent flies; lines represent means (no ExR2 activation, NS, *P* = 0.34; ExR2 activation, **P = 0.0039; ExR2 activation with no cue, NS, *P* = 0.22; two-sided one-sample *t*-tests with Bonferroni correction). **g**, Change in the mean offset between the bump position and the cue position (post − pre), measured in the windows shown in **c**. Dots represent flies; lines represent means (NS, *P* = 0.70 in both cases, Kruskal–Wallis tests).

The arthropod head direction system is laid out as a circular topographic map^27,34^, unlike the mammalian head direction system. In both cases, however, the assignment of visual cues to head direction neurons is flexible and seemingly arbitrary. We wondered how dopamine reorganizes this topographic map. To answer this question, we carried out two-photon imaging of jGCaMP7f in the entire EPG ensemble (Fig. 3a). As before, we rotated a visual cue at a constant speed while transiently stimulating ExR2 dopamine neurons. At the outset of a typical experiment, we observed a discrete bump of activity that rotated around the topographic map of head direction in sync with the visual cue (Fig. 3b, top). Often, however, the bump did not track the cue accurately, probably because the imposed rotation of the cue conflicts with the fly’s internal self-motion signals (that is, its motor commands and proprioceptive feedback). Transiently stimulating ExR2 dopamine neurons persistently increased the accuracy of visual cue tracking in the EPG ensemble (Fig. 3b). We quantified this by estimating the mutual information that the EPG bump position conveyed about the position of the visual cue; mutual information increased after ExR2 stimulation and remained high for the rest of the experiment (>10 min; Fig. 3c,d). This result indicates that a burst of dopamine neuron activity can persistently strengthen the influence of a visual cue on head direction neurons. We also observed that ExR2 activation increased the bump amplitude, although this effect was more transient (Fig. 3e,f), mirroring the relatively transient increase we observed in single-cell visual response amplitude (Fig. 2e,f). None of these effects occurred in control experiments in which ATP was washed into the bath but ExR2 neurons did not express P2X2 receptors (Fig. 3c–f).

Notably, we found that ExR2 stimulation did not rotate the coordinate frame of the entire head direction map: the overall ‘offset’ of the EPG bump relative to the visual cue was equally stable over time in experimental versus genetic control flies (Fig. 3g). Rather, the map simply became more self-consistent, so that there was now a more orderly one-to-one correspondence between cue positions and bump positions. To obtain a more orderly map, it is logical that some neurons must shift their preferences, and so it makes sense that we observed some instances of clear cue-preference shifts in our single-cell recordings (Fig. 2b,c).

Next, we asked whether these changes require dopamine to be paired with the visual cue. We repeated our experimental protocol, but now we turned off the visual cue to stimulate ExR2 neurons in darkness (Fig. 3b, bottom). At the time when ExR2 firing rates should have returned to baseline, we turned the visual cue back on and retested its effect on the EPG bump position. Here we found no increase in the mutual information between the bump position and the cue position (Fig. 3c,d). In darkness, we did observe a transient increase in EPG bump amplitude, but this effect did not outlast the time when ExR2 firing rate would be elevated (Fig. 3e,f). Thus, dopamine must be paired with the cue; otherwise, it does not strengthen the subsequent influence of the cue.

## Decreasing dopamine release

**Fig. 4:**
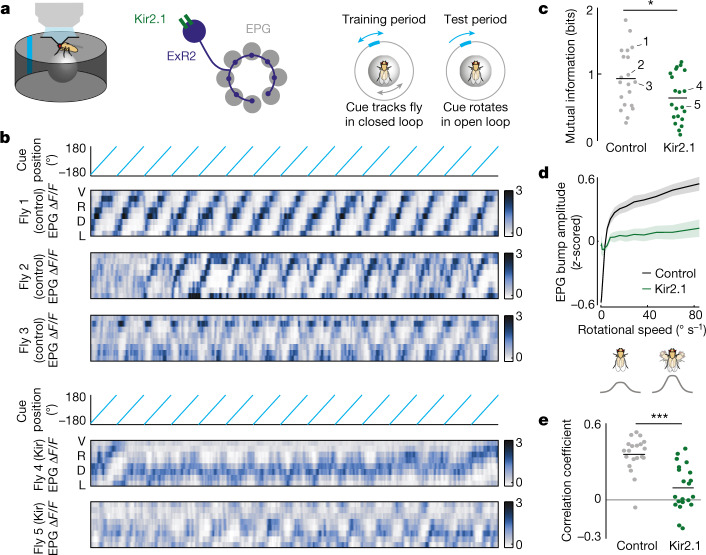
Suppressing ExR2 activity reduces the influence of a visual cue on the head direction map. **a**, Imaging jGCaMP7f in EPG neurons while ExR2 activity is suppressed through Kir2.1 expression. In the training period, the visual cue was controlled by the fly’s rotation on the spherical treadmill. In the test period, the cue was rotated at a constant velocity. **b**, Example test period data for three control flies (top) and two Kir2.1 flies (bottom). In each Δ*F*/*F* heatmap, adjacent rows are adjacent wedges of the circular map in the ellipsoid body. **c**, Estimate of the mutual information between the cue position and the bump position in the test period. Dots represent flies; lines represent means (*n* = 21 with Kir2.1 expression; *n* = 20 control here and in **e**); examples in **b** are labelled. Mutual information is lower in the Kir2.1 flies (*P = 0.027, two-sided two-sample *t*-test). **d**, EPG bump amplitude versus the fly’s rotational speed for both genotypes; data are binned by speed and averaged within a fly before averaging across flies (±s.e.m. across flies). Only speeds ≤100° s^−1^ are included. Bump amplitude is *z*-scored within each fly. The schematic shows higher bump amplitude with higher rotational speed. **e**, Correlation between bump amplitude and rotational speed throughout the experiment. Dots represent flies; lines represent means. In flies in which ExR2 neurons express Kir2.1, this correlation is lower (***P = 3.5 × 10^−6^, two-sided two-sample *t*-test with Fisher transformation).

Finally, we asked whether decreasing dopamine release can interfere with cue associations. We expressed an inwardly rectifying potassium channel (Kir2.1) in ExR2 neurons to hyperpolarize these cells, whereas in control flies, we omitted the ExR2 Gal4 line, so that Kir2.1 was not expressed. In both genotypes, jGCaMP7f was expressed in EPG neurons under LexA control. In the first block of each experiment, we allowed the fly to walk in a virtual reality environment containing a visual cue that rotated around the fly whenever the fly turned on the spherical treadmill (Fig. 4a). This closed-loop ‘training’ block should allow Hebbian strengthening of the association between the cue and the bump, and normal ExR2 activity should potentiate cue–bump associations. Subsequently, in the second block of each experiment, we tested the cue’s influence on the bump: we rotated the cue around the fly at a constant speed, decoupled from the fly’s walking. During this ‘test’ block, we would predict that the influence of the cue will be lower if ExR2 dopamine release has been suppressed during the training period.

**Extended Data Fig. 9:**
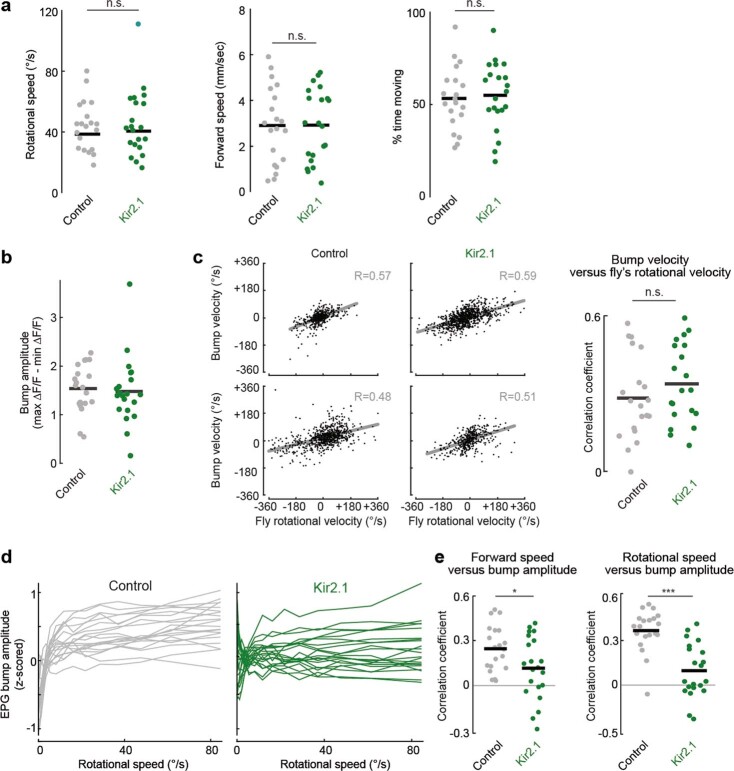
Effects of hyperpolarizing ExR2 dopamine neurons. a) Mean rotational speed (left), forward speed (center), and percentage of time spent locomoting (right) throughout the experiment, for both the control genotype and the genotype where ExR2 neurons express Kir2.1 (n = 20 control and 21 Kir2.1 flies throughout this figure). Dots are individual flies; horizontal lines are means. There was no significant difference between the two genotypes for any of these locomotor parameters (p = 0.81, p = 0.97, and p = 0.79, two-sided Wilcoxon rank sum test). In short, we find no evidence that ExR2 neuron hyperpolarization causes locomotor defects. b) EPG bump amplitude was not significantly different in the genotype where ExR2 neurons express Kir2.1 versus the control genotype (p = 0.76, two-sided two-sample t-test). c) *Left:* Data from two example flies from each genotype, showing a similar correlation between the bump rotational velocity and the fly rotational velocity. This result implies that hyperpolarizing ExR2 neurons does not impair the tendency of the bump to track the fly’s internal self-motion signals. Each data point is a time point. *Right:* Pearson’s correlation coefficient between bump rotational velocity and fly rotational velocity, for all flies. Note that these correlations are all relatively low, because they were all measured as the visual cue was rotating in open loop around the fly, and so internal self-motion signals were competing with visual cues for control of the bump. The visual cue has less influence when ExR2 neurons express Kir2.1 (Fig. 4c), and so it not surprising that there is a trend toward a larger relative influence of self-motion signals in the Kir2.1 flies (i.e., a slightly stronger correlation with the fly’s rotational velocity), although this trend was not significant (p = 0.28, two-sided two-sample t-test, n = 20 Kir2.1 flies and 20 control flies). The two example flies from each genotype both have relatively high correlations, but they are typical in having a fitted slope less than unity; this has been observed previously in flies walking in darkness^27^, and it suggests that the native gain of the EPG network is <1, i.e. the brain tends to underestimate the fly’s rotation on the spherical treadmill. d) EPG bump amplitude versus the fly’s rotational speed for both genotypes; data are binned by speed and averaged within a fly. Bump amplitudes are z-scored. Each line is an individual fly. Fig. 4D shows the mean±SEM within each genotype. e) *Left:* Pearson’s correlation coefficient between bump amplitude and forward speed throughout the experiment. Dots are flies; lines are means. In flies where ExR2 neurons express Kir2.1, this correlation is lower (p = 0.029, two sample t-test with Fisher transformation). *Right:* same but for rotational speed (p = 3.5 × 10^−6^, two-sided two sample t-test with Fisher transformation). This plot is reproduced from Fig. 4e for comparison. Note that the magnitude of correlation coefficient is stronger for rotational speed than for forward speed. Because forward speed and rotational speed are themselves correlated (Extended Data Fig. 2b), it is expected that bump amplitude would be at least weakly correlated with forward speed.

Our results confirmed this prediction. The influence of the visual cue during the test block was indeed weaker in ExR2-hyperpolarized flies versus control flies (Fig. 4b), as evidenced by the significantly lower mutual information between the cue position and the bump position (Fig. 4c). This finding implies that ExR2 activity is required for the normal formation of a visual cue association in EPG neurons. Notably, ExR2 hyperpolarization had no effects on the fly’s walking behaviour, and it did not impair the bump’s ability to track the fly’s internal self-motion signals (Extended Data Fig. 9).

Recall that ExR2 activity scales with the fly’s rotational speed (Fig. 1c,d), and ExR2 activity transiently increases EPG bump amplitude (Figs. 2f and 3f). We found that bump amplitude is also correlated with the fly’s rotational speed (Fig. 4d), confirming the findings of previous reports^27,35–37^. Notably, we found that hyperpolarizing ExR2 neurons almost completely eliminated the correlation between bump amplitude and rotational speed (Fig. 4d,e). Thus, speed-evoked bump amplitude changes are at least partly due to dopamine release.

## Discussion

In summary, we find that ExR2 dopamine neuron activity promotes associations between sensory cues and head direction cells. These associations have been shown to reorganize when the fly enters a new environment with a new cue configuration, probably through Hebbian plasticity at ER-to-EPG synapses^28,29^. In the *Drosophila* mushroom body, dopamine can promote both synaptic potentiation and synaptic depression by acting on different dopamine receptor types^20,38,39^. ER neurons express multiple dopamine receptor types^24,26,40,41^, as do EPG neurons^42^. ER-to-EPG synapses are in close proximity to dopamine release sites (Extended Data Fig. 1). In the future, it will be interesting to determine the roles of different dopamine receptors in ER-to-EPG synaptic plasticity. Notably, a study published while our work was in revision^26^ reported that multiple dopamine receptors in ER neurons are required for a fly to learn to steer away from a direction associated with punishment; that study also showed that hyperpolarizing ExR2 neurons blocks this form of learning. Our results extend this work by demonstrating that flies do not form normal associations between visual cues and head directions when ExR2 neurons are hyperpolarized. These flies have an impairment in mapping visual cue locations onto their internal map of head direction, and so it is logical that they would be unable to use visual cues to steer in an arbitrary (remembered) direction to avoid a predicted punishment.

Notably, ExR2 dopamine neurons are primarily active when the fly is rotating. This is probably partly due to the synaptic inputs to ExR2 neurons in the lateral accessory lobe, a brain region where many descending neurons are located^43^, including descending neurons involved in steering^30^. Moreover, ExR2 neurons also receive inputs from PEN_a neurons that track rotational movements^35,36^. Rotational movements have a special relevance for the head direction system: they coincide with internal cues (motor commands and proprioceptive signals) that can be compared with the displacements of external head direction cues, including visual cues, sky-wide visual patterns^34^ and the direction of the prevailing wind^44–46^. As such, rotational movements provide a rich dataset for statistical learning in the head direction system. Our results argue that dopamine serves to compress learning into these data-rich epochs. Notably, certain dopamine neurons in the mammalian brain are time-locked to specific kinematic features of motor performance^15,16,18^. It is tempting to speculate that these mammalian dopamine neurons serve a similar function, by compressing synaptic plasticity into the time intervals when task-specific movements are generating rich datasets for learning.

It is worth asking why the system does not learn continuously. One of the behavioural functions of the head direction system in *Drosophila*^47,48^ and other arthropods^49,50^ is to enforce a straight-line path while navigating towards a distant goal destination. However, straight-line locomotion will stabilize the visual world on the retina, and so if the system were learning continuously, then the brain’s map of head direction would become progressively skewed, as the current view is over-learned. Thus, there is an inherent tension between goal-directed action, which limits the range of experiences, and statistical learning, which requires broad sampling of experiences. One solution to this problem is to pre-memorize a snapshot of the world before starting to pursue a goal—as dung beetles do when they rotate in place before embarking on a trip^51^. Then, during goal-directed navigation, the brain should compress any further orientation learning into moments of active reorienting behaviour. This is reminiscent of machine learning protocols, in which learning and performance are typically partitioned into separate epochs^4^.

In machine learning, it is conventional to draw a distinction between reinforcement learning (learning through trial and error to choose actions in a manner that maximizes reward) and unsupervised learning (inferring the structure of a dataset without the benefit of rewards or labels). In the brain, dopamine has a clear role in reinforcement learning^6^. By contrast, dopamine’s potential role in unsupervised learning is less well understood. Spatial learning is a prime example of unsupervised learning: here, the brain’s task is simply to infer the structure of the environment through exploration. Our results reveal a link between dopamine and spatial learning, highlighting the need for a ‘supervisory’ element that controls the rate of the (otherwise) unsupervised inference.

## Methods

### Cell types and synonyms

We have adopted the cell type names used in the recent comprehensive anatomical survey of the central complex^25^. ExR2 neurons are the dopamine neurons of the PPM3 cluster that innervate the ellipsoid body^52^ (that is, PPM3-EB neurons^53^). EPG neurons have also been termed E-PG or PBG1-8.b-EBw.s-D/Vgall.b neurons^54,55^, EB-IDFP D/VSB-PB neurons^56^ or compass neurons^36^. ER neurons^57^ have been termed ring neurons or R neurons^58^, as well as LTR-EB neurons^59^. PEN_a neurons are also referred to as P-EN1 (ref. ^35^) or PEN1 (ref. ^57^).

### Fly husbandry and genotypes

Unless otherwise stated, flies were raised on standard cornmeal–molasses food (New Brown 19L, Archon Scientific) in an incubator on a 12/12-h light/dark cycle at 25 °C with humidity about 50–70%. All experiments used flies with at least one wild-type copy of the *white* gene, and many experiments used flies with two wild-type copies of the *white* gene (as detailed below). The experimenters were not blind to genotype. In Fig. 2, flies of the appropriate genotype and age were selected randomly and then alternately assigned to the ‘ExR2 activation’ condition or the ‘ExR2 activation, no visual cue’ condition, so that the two conditions were interleaved in the data acquisition sequence; these assignments were made prior to the beginning of each experiment.

The genotypes of the fly stocks used in Fig. 1 and Extended Data Figs. 2–4 are as follows: *+/+* or +/w*; P{75B10-LexA}attP40/P{R75B10-LexA}attP40; PBac{13×LexAop-IVS-jGCaMP7f}VK00005/PBac{13×LexAop-IVS-jGCaMP7f}VK00005.

The genotype of the fly stock used in Fig. 2a is as follows: *+/+; P{R38A11-LexA}attP40/P{13×LexAop2-mCD8::GFP}attP40; P{13×LexAop2-mCD8::GFP}attP2/P{LexAop-P2X2.Y}3*.

The genotypes of the fly stocks used in Fig. 2b–f and Extended Data Figs. 6 and 7 are as follows: for control, *+/+* or +/w*; R38A11-LexA/UAS-mCD8::GFP; R60D05-Gal4/+; for ExR2 activation, *+/+* or +/w*; R38A11-LexA/UAS-mCD8::GFP; LexAop-P2X2.Y/R60D05-Gal4; for dopamine, *+/+; UAS-mCD8::GFP/UAS-mCD8::GFP; R60D05-Gal4/R60D05-Gal4* (*n* = 9) or *+/+* or +/w*; R38A11-LexA/UAS-mCD8::GFP; R60D05-Gal4/+ (*n* = 3).

The genotypes of the fly stocks used in Extended Data Fig. 8 are as follows: for no Chrimson control, *+/+; P{20XUAS-IVS-mCD8::GFP}attP40, PBac{13×LexAop2-IVS-Syn21-Chrimson::tdT-3.1-p10} su(Hw)attP5/+; P{R60D05-Gal4}attP2/+*; for ExR2 activation and ‘no cue’ conditions, *+/+; P{20XUAS-IVS-mCD8::GFP}attP40, PBac{13×LexAop2-IVS-Syn21-Chrimson::tdT-3.1-p10} su(Hw)attP5/P{R38All-LexA}attP40; P{R60D05-Gal4}attP2/+.*

The genotypes of the fly stocks used in Fig. 3 are as follows: for control, *+/+* or +/w*; P{R38A11-LexA}attP40/P{20XUAS-IVS-jGCaMP7f}su(Hw)attP5; P{R60D05-Gal4}attP2/+; for ExR2 activation, *+/+* or +/w*; P{R38A11-LexA}attP40/P{20XUAS-IVS-jGCaMP7f}su(Hw)attP5; P{LexAop-P2X2.Y}3/P{R60D05-Gal4}attP2.

The genotypes of the fly stocks used in Fig. 4 and Extended Data Fig. 9 are as follows: for control, w[1118], *P{13×LexAop-IVS-jGCaMP7f}su(Hw)attP8/+; P{R60D05-LexA}attP40/+; P{UAS-Hsap\KCNJ2.eGFP}7/+;* for Kir2.1, w[1118], *P{13×LexAop-IVS-jGCaMP7f}su(Hw)attP8/+; P{R60D05-LexA}attP40/+; P{UAS-Hsap\KCNJ2.eGFP}7/P{R75B10-Gal4}attP2*.

### Characterization and description of driver line expression

We used *P{R60D05-Gal4}attP2 or P{R60D05-LexA}attP40* to target EPG neurons for calcium imaging or electrophysiology recording as established in previous studies^27,28^.

We used *P{R38A11-LexA}attP40* to drive expression of mCD8::GFP and P2X2 receptors in ExR2 neurons. Immunostaining of these brains showed that GFP expression was isolated to four ExR2 neurons, a bilateral pair of unidentified ascending neurons that arborize within the antennal lobe, and 1–2 pars intercerebralis neurons (data not shown). The database of the FlyLight Project Team at Janelia Research Campus (https://flweb.janelia.org/cgi-bin/flew.cgi) showed a similar expression pattern for this line.

We used *P{R75B10-LexA}attP40* to carry out calcium imaging from ExR2 neurons because *R38A11-LexA* produces relatively weak jGCaMP7f expression, and we obtained a better signal-to-noise ratio in two-photon imaging when we used *R75B10-LexA* to drive jGCaMP7f expression. Images from the FlyLight database (https://flweb.janelia.org/cgi-bin/flew.cgi) show that this line has strong expression in a subset of dopamine neurons from the PPM3 cluster, namely ExR2 FB4M and/or FB4L. This line also has weaker expression in a lobula cell type, the SEZ, and the SMP. During calcium imaging, the only detectable jGCaMP7f expression in the ellipsoid body and bulb regions was from the ExR2 neurons. We observed that jGCaMP7f signals were highly correlated in the ellipsoid body and bulb, as we would expect if these signals arose from ExR2 neurons exclusively.

We used *P{R75B10-Gal4}attP2* to drive expression of Kir2.1 in ExR2 neurons. Images from the FlyLight database (https://flweb.janelia.org/cgi-bin/flew.cgi) show that this line has strong expression in ExR2 dopamine neurons in the ellipsoid body, as well as some labelling in a middle layer of the fan-shaped body. This line also has weaker expression in a lobula cell type, the SEZ, and the SMP. Immunostaining of GFP protein in flies with the genotype *w[1118], P{13×LexAop-IVS-jGCaMP7f}su(Hw)attP8 / +; P{R60D05-LexA}attP40 / +; P{UAS-Hsap\KCNJ2.EGFP}7 / P{R75B10-Gal4}attP2* revealed the clearest Kir2.1::eGFP expression in the ExR2 neuron and middle fan-shaped body layers.

### Origins of transgenic stocks

*P{20XUAS-IVS-mCD8::GFP}attP40* and *PBac{13×LexAop2-IVS-Syn21-Chrimson::tdT-3.1-p10}su(Hw)attP5* were gifts from B. Pfeiffer and G. Rubin and have previously been published^60,61^. Rubin Gal4 and LexA lines (*P{R60D05-Gal4}attP2*, *P{R38A11-LexA}attP40, P{R75B10-LexA}attP40* and *P{R75B10-Gal4}attP2*) were obtained from the Bloomington *Drosophila* Stock Center (BDSC); the general methods for constructing these lines have previously been published^60,62,63^. Lines for expressing jGCaMP7f under LexAop or UAS control (*PBac{13×LexAop-IVS-jGCaMP7f}VK00005* and *P{20XUAS-IVS-jGCaMP7f }su(Hw)attP5*) were obtained from the BDSC and have previously been published^37^. Lines for expressing GFP under LexAop control (*P{13×LexAop2-mCD8::GFP}attP40* and *P{13×LexAop2-mCD8::GFP}attP2*) were obtained from the BDSC and have previously been published^60^. The line used for expressing P2X2 under LexAop control (*P{LexAop-P2X2.Y}3*) was obtained from the BDSC and has previously been published^64^. The line for expressing Kir2.1::eGFP under UAS control (*P{UAS-Hsap\KCNJ2.eGFP}7*) was obtained from the BDSC and has previously been published^65^.

### Fly preparation and dissection

Newly eclosed female *Drosophila melanogaster* were anaesthetized on ice and were collected about 3–10 h (electrophysiology) or 1–4 days (imaging) before the experiment. For the ExR2 imaging experiments in Fig. 1, we deprived the flies of food (but not water) and kept them in isolation for approximately 24 h before the experiment to promote walking behaviour.

For electrophysiology experiments, the fly holder consisted of flat titanium foil secured within an acrylic platform. The fly’s head was pitched forwards so that the posterior surface was parallel to the foil and most of each eye was under the foil. For imaging experiments, a holder was custom designed using CAD software (OnShape) and created in-house using a three-dimensional (3D) printer (Form 2 with Grey Pro and Rigid resins, Formlabs) to expose a larger surface area of the fly’s eye below the holder, and the fly’s head was pitched only slightly forwards (<30°).

The fly was secured in the holder using ultraviolet-curable adhesive (Loctite AA 3972) cured by a brief (<1 s) pulse of ultraviolet light (LED-200, Electro-Lite Co.). After the dorsal portion of the head (above the holder surface) was covered in saline solution, a hole was cut in the head capsule and trachea were removed to expose the posterior surface of the brain. To reduce brain movement, muscle 16 and proboscis muscles were clipped with forceps. For electrophysiology, an aperture was made in the perineural sheath by pulling gently with fine forceps or by using suction from a patch pipette containing external solution.

The external saline solution contained (in mM): 103 NaCl, 3 KCl, 5 *N*-tris(hydroxymethyl) methyl-2-aminoethane-sulfonic acid, 8 trehalose, 10 glucose, 26 NaHCO_3_, 1 NaH_2_PO_4_, 1.5 CaCl_2_ and 4 MgCl_2_, with osmolarity adjusted to 270–273 mOsm. External solution was bubbled with 95% O_2_ and 5% CO_2_ and reached a final pH of 7.3. The external saline solution was continuously perfused over the brain during experiments.

### Patch-clamp recordings

Patch pipettes were made from borosilicate glass (Sutter, 1.5 mm o.d., 86 mm i.d.) using a Sutter P-97 puller. Pipettes were fire polished down after pulling^66^ using a microforge (ALA Scientific Instruments) to a final resistance of 8–15 MΩ. The internal pipette solution contained (in mM): 140 potassium aspartate, 10 4-(2-hydroxyethyl)-1-piperazineethanesulfonic acid, 4 MgATP, 0.5 Na_3_GTP, 1 ethylene glycol tetraacetic acid, 1 KCl and 13 biocytin hydrazide. The pH was 7.3, and the osmolarity was adjusted to about 268 mOsm. For experiments that did not require rapid pharmacology, the saline solution was perfused at about 2 ml min^−1^; higher flow rates were used for rapid drug delivery (see below). Recordings were carried out at room temperature.

To visualize the brain, we used a custom-modified Olympus BX51WI microscope with a 40× water-immersion objective. We removed the light source and condenser below the preparation, and we instead illuminated the brain with far-red light delivered by a fibre-coupled light-emitting diode (LED; 740 nm, M740F2, Thorlabs) through a ferrule patch cable (200 µm Core, Thorlabs) plugged into a fibre optic cannula (1.25-mm SS ferrule 200-µm core, 0.22 NA, Thorlabs) glued to the recording platform, with the tip of the cannula about 1 cm behind the fly. GFP fluorescence was visualized using a mercury arc lamp (U-LH100HG, Olympus) with an eGFP-long-pass filter (U-N41012, Chroma).

Somatic recordings were obtained in current-clamp mode using an Axopatch 200B amplifier and a CV-203BU headstage (Molecular Devices). Voltage signals were low-pass filtered at 5 kHz before digitization and then acquired with a NiDAQ PCI-6251 (National Instruments) at 20 kHz. To counteract the depolarizing leak current caused by the finite resistance of the patch electrode seal^67^, we applied a constant hyperpolarizing current throughout the experiment that lowered the somatic membrane potential by about 5 mV. For all electrophysiological recordings, liquid junction potential correction was carried out post hoc by subtracting 13 mV from recorded voltages^67^.

### Two-photon calcium imaging

Imaging experiments were carried out as previously described^28^ using a two-photon microscope with a movable stage (Thorlabs Bergamo II) and a fast piezoelectric objective scanner (Physik Instrumente P725) for volumetric imaging. We used a Chameleon Vision-S Ti-sapphire femtosecond laser tuned to 940 nm for two-photon excitation. Images were collected using a 20× 0.95-NA objective (Olympus). Emission fluorescence was filtered with a 525-nm bandpass filter (Thorlabs) and collected using a GaAsP photomultiplier tube (Hamamatsu).

For EPG neurons, the imaging region was centred on the protocerebral bridge, where EPG axons terminate. The imaging view was 256 × 128 pixels, and 8–12 slices deep in the *z* axis (4-6 µm per slice), resulting in a 6–9 Hz volumetric scanning rate. For ExR2 neurons, the imaging region was centred on the bulb and ellipsoid body. The imaging view was 256 × 128 pixels, and 12 slices deep in the *z* axis (6-8 µm per slice), resulting in a 6–7 Hz volumetric scanning rate.

Volumetric *z*-scanning signals from the piezoelectric objective scanner were acquired simultaneously with analog output signals from the visual panorama and/or analog outputs from FicTrac 2.1 through a NiDAQ PCI-6341 at 40 kHz. Data were acquired using ScanImage 2018 (Vidrio Technologies) with National Instruments hardware from Vidrio (NI PXIe-6341).

### Measuring locomotor behaviour

For the experiments in Figs. 1 and 4, the fly stood on a spherical treadmill, which was a 9-mm-diameter ball made of white foam (FR-4615, General Plastics) painted with black shapes. The ball floated above a 3D printed plenum made of clear acrylic (Autotiv). Medical-grade breathing air flowed into the base of the plenum and flowed out into a hemispherical depression that cradled the ball to allow it to rotate freely. The ball was illuminated from below by two round boards of 36 IR red LED lamps (SODIAL). The movement of the ball was tracked at about 60 Hz using a video camera (CM-3-U3-13S2M-CS, FLIR) fitted with a Tamron 23FM08L 8-mm 1:1.4 macro zoom lens. Machine vision software (FicTrac 2.1) was used to convert the image of the ball to a running estimate of the ball’s position in all three axes of rotation^68^. The fly’s rotational velocity was inferred from the yaw velocity of the ball. The fly’s forward velocity was inferred from the pitch velocity of the ball. The fly’s fictive heading direction (head direction) was inferred from the temporal integral of the fly’s rotational velocity.

### Pharmacology

For pharmacology experiments, external saline solution was perfused quickly using a Watson Marlow Pump (120U) set at 60 r.p.m. (about 5 ml min^−1^). At the start of a treatment trial, the intake tube was moved for exactly 30 s (electrophysiology) or 60 s (imaging) from normal saline solution into saline containing dopamine (200 µM) or ATP (5 mM). On each rig, we carried out measurements of the time it takes a new solution to flow through the tubing and reach the recording chamber. For electrophysiology, we validated this tubing delay time estimate by placing a recording electrode in the bath in voltage-clamp mode and then perfusing in external solution with a much higher salt content (>1 M NaCl) in the exact same manner that ATP or dopamine solutions were normally delivered. The first deviation in current signal measured by the electrode was used to estimate the entry of the new solution. For data display, measurements are plotted relative to the earliest time when the drug (ATP or dopamine) entered the recording chamber (*t* = 0).

We prepared the ATP solution (5 mM in bubbled saline) immediately before application from a frozen stock of 500 mM ATP in water. We prepared the dopamine solution (200 µM dopamine + 100 µM sodium metabisulfite to minimize oxidation^69^, prepared in saline) on a daily basis, using a frozen stock of 100 mM dopamine in 50 mM sodium metabisulfite in water. ATP solutions and dopamine solutions were bubbled with 95% O_2_ and 5% CO_2_ before application.

To verify the timing of ExR2 activation using ATP, we carried out whole-cell current-clamp recordings from ExR2 neurons (Fig. 2a), using the same ATP protocol, the same ExR2 LexA driver line and the same LexAop-P2X2 effector transgenes that we used in our EPG electrophysiology experiments. This comparison was only possible in electrophysiology experiments because the highly specific ExR2 driver line used to drive P2X2 expression did not drive enough jGCaMP7f expression for high-quality two-photon imaging from ExR2 neurons.

### Chrimson stimulation

Flies expressing Chrimson^70^ were raised for all of development on Nutri-Fly “German Food” Sick Fly Formulation no. 66-115 (Genesee) containing about 0.6 mM all-*trans* retinal (Sigma) and tegosept anti-fungal agent. Fly vials were wrapped in foil to prevent photo-conversion of the all-*trans* retinal. For optogenetic stimulation, we used the Hg-lamp source (U-LH100HG) to deliver pulses of orange light (590–650 nm, 2–3 mW, Cy5 long-pass filter cube (49019, Chroma) through the objective. A shutter (Uniblitz Electronic) controlled the pulse pattern (0.5 s on, 1 s off) that was paired with the visual presentation of a bright vertical bar that rotated around the fly in the same manner as described below for all other electrophysiology experiments.

### Visual panorama

Visual stimuli were presented to the fly using a circular panorama (IORodeo) made of modular square panels^71^ as previously described^28^. Each square panel was made up of an 8 × 8 array of LEDs (8 × 8 ‘pixels’) that refresh at 372 Hz or faster^71^. For electrophysiology experiments, these LEDs were green (LED peak = 525 nm). In imaging experiments, these LEDs were blue (LED peak = 470 nm) to minimize overlap with GCaMP emission spectra.

For electrophysiology experiments, we used a panorama that spanned 270° in azimuth; the panorama covered the azimuthal range from about 124° left of the midline to about 147° right of the midline (that is, it was slightly asymmetric). For imaging experiments, we used a 360° panorama, with one square panel behind the fly removed for camera positioning. The visual panoramas spanned about 43° vertically of the fly’s visual field and a single LED pixel along the top of the arena subtended about 3.6–3.7° of the fly’s visual field.

In electrophysiology experiments, to reduce electrical noise, the panorama was wrapped with grounded copper mesh. To reduce reflections, the mesh was covered with black ink, and the front surface of each panel was covered with a diffuser (SXF-0600 Snow White Light Diffuser, Decorative Films). In imaging experiments, five layers of filters (Rosco R381, bandpass centre 440, full-width at half-maximum 40 nm) were placed in front of the panels to minimize detection of the visual stimulus by the GCaMP emission collection channel. Analog output signals from the visual panel system were digitized with a NiDAQ PCI-6251 (National Instruments) at 20 kHz (electrophysiology) or with a NiDAQ PCI-6341 (National Instruments) at 40 kHz (calcium imaging).

### Visual stimuli

The visual cue was a bright vertical bar (2 pixels wide, 7°) that spanned the full height of the panorama (about 43°). For open-loop trials, the bar was rotated continuously around the fly at about 18° s^−1^ in the rightward direction. In imaging experiments with the 360° arena, the top half of the bar was ‘jumped’ over the missing panel directly behind the fly to maintain a constant total luminance within the arena at all times. For the closed-loop training period described in Fig. 4, the angular position voltage signal from FicTrac 2.1 was used to continuously update the azimuthal position of the visual cue displayed on the panorama. Thus, when the ball rotated rightwards (indicating an attempted leftward rotational manoeuvre by the fly), the visual cue rotated rightwards at the same velocity. For the optic flow stimulus in Fig. 1f, we presented a 360° vertical grating consisting of alternating 7° dark and light stripes. The grating appeared on the screen and remained static for 4 s before starting a about 18° s^−1^ leftward or rightward rotation, to isolate responses to the optic flow from any responses to the appearance of the grating. On average, the appearance of the vertical grating produced a relatively small transient ExR2 response (Δ*F*/*F* ≈ 0.4, not shown). The grating rotated for 4 s before disappearing, and Δ*F*/*F* responses were measured in the last 2 s of this stimulus period. The analog output signals from the visual panel system and from FicTrac 2.1 were digitized with a NiDAQ PCI-6341 (National Instruments) at 40 kHz. A visual cue position of 0° means the cue is directly in front of the fly.

### Connectome analysis

To analyse the proximity of ExR2 output sites to ER-to-EPG neuron synapses, we analysed a partial connectome of the dorsal part of the right central brain of an adult female fly obtained by the FlyEM project at Janelia Research Campus (https://janelia.org/project-team/flyem/hemibrain)^57^. Analysis was carried out in R using the neuprintr extension^72^ of neuprint^73^. ExR2, ER and EPG annotations were taken from the hemibrain v1.2.1 release. We calculated the Euclidean distance between each ER-to-EPG neuron pre-synapse and the ExR2 release site that is closest to that pre-synapse. Analysis was restricted to synapses within the ellipsoid body neuropil region. The boundaries of the ellipsoid body and other neuropil regions (Extended Data Fig. 1) were extracted from the hemibrain dataset.

Our analysis of mushroom body synapses (Extended Data Fig. 1) focused on Kenyon cells (KCs), mushroom body output neurons (MBONs) and mushroom body dopamine neurons (MB-DANs). We calculated the Euclidean distance between each KC-to-MBON synapse and its closest MB-DAN output synapse. This analysis was restricted to neurons on the right side of the brain with clear compartmentalization in the gamma lobe of the mushroom body. Our results of mushroom body synapses are consistent with those of a previous analysis^74^.

### Data analysis

Data analysis was carried out using Matlab R2016b, R2017a, R2017b, 2019b, R2020b (MathWorks), Python 3.9.5, R 4.1.0 and RStudio 1.4.1717. Throughout the figures, statistical tests are summarized as follows: NS, *P* > 0.05; *P < 0.05; **P < 0.01; ***P < 0.0005.

#### Calcium imaging alignment and data processing

Rigid motion correction in the *x* and *y* axes was carried out for each acquisition using the NoRMCorre algorithm^75^. Volumetric regions of interest (ROIs) were defined by combining 2D ROIs drawn in multiple imaging planes. Fluorescence values for each ROI were determined by averaging all pixels in that volumetric ROI. All fluorescence data were smoothed with a Gaussian kernel 600 ms in width before analysis. For ExR2 imaging (Fig. 1), ROIs were drawn throughout the ellipsoid body (EB) and the left and right bulbs. ROIs from the EB and the left and right bulbs were combined for analysis to improve the signal-to-noise ratio. For EPG imaging, 16 volumetric ROIs corresponding to the 16 glomeruli in the protocerebral bridge were defined on the basis of visible anatomical boundaries. ROIs of EPG neurons from the left and right hemispheres that occupy the same part of the EB were then combined to create one ROI for each of the eight EB wedges. To calculate the time-dependent change in fluorescence (Δ*F*/*F*) for each ROI, we used a baseline fluorescence (*F*) defined as the fifth percentile of raw fluorescence values for that ROI throughout the entire experiment. For the optic flow analysis in Fig. 1f, fluorescence in an empty background ROI was subtracted from fluorescence in the ExR2 ROI, to adjust for any light from the visual panels that was picked up by the photomultiplier tube during imaging. For that analysis, Δ*F*/*F* was calculated using the mean fluorescence from the 2 s before the stimulus onset as the baseline *F*.

#### ExR2 locomotor correlations

The displacement of the spherical treadmill was computed by FicTrac 2.1 (ref. ^68^) in each of the three axes of rotation at about 60 Hz. This was then used to calculate forward and rotational speeds of the fly at each time point. Speed data were downsampled to match the volume rate of the imaging data and smoothed 10 times with a Gaussian kernel 60 ms in width. Finally, the speed data were shifted back in time by two imaging volumes (about 300 ms), because this maximized the correlation between the two signals. In the example traces shown in Fig. 1b, the speed traces were smoothed one additional time with a Gaussian kernel 600 ms in width after downsampling and were not shifted in time. For the binned rotational speed analysis (Fig. 1c), all of the Δ*F*/*F* data for a given experiment were divided into 45 speed bins, and the mean Δ*F*/*F* within each bin was calculated. Bin widths and edges were chosen so that each bin contained the same number of data points. For the binned 2D speed analysis (Fig. 1d), speed and Δ*F*/*F* from all experiments were combined and then divided into bins on the basis of 2D speed. The bins in this analysis had a uniform size in each speed axis, meaning that the number of data points per bin was not uniform across bins. The grey shading in Fig. 1d shows the range of very low rotational speeds that occur during transitions between resting and moving; all of these speeds (including rest periods and rest–move transitions) were included in our analyses. In a subset of experiments, the same analysis was carried out on trials in which the visual cue was rotated around the fly in an open loop (Extended Data Fig. 4b), and for separate experiments the equivalent analysis was carried out with the same cue in a closed loop (Extended Data Fig. 4a). A line was fitted to the portion of each of the resulting curves with rotational speeds greater than 30° s^−1^ using Matlab’s fitlm() function. To generate the plots in Extended Data Fig. 3a, ROIs were drawn around ExR2 neurites in the left and right lateral accessory lobes, and Δ*F*/*F* was calculated as described above. Correlation coefficients between the fly’s rotational speed and ExR2 activity in each ROI were calculated using Matlab’s corrcoef() function. Only epochs in which the fly was moving (rotational speed >15° s^−1^ or forward speed >2 mm s^−1^) were included in the analysis. The plots in Extended Data Fig. 3b were generated using data from two different 5-min epochs during a single recording, with a 5-min gap between them. The correlation between rotational speed and ExR2 activity was calculated as described above, and the resulting coefficients were smoothed with a 2D Gaussian kernel (σ = 1.5) and manually thresholded to show only the pixels with the strongest positive and negative correlations. Background (greyscale) images in Extended Data Fig. 3b show trial-averaged fluorescence from these same five imaging planes.

#### Linear model analysis

Forward and rotational speed data were processed as described above, and then *z*-scored and used as predictor variables. Fluorescence data were *z*-scored and used as the dependent variable. For each experiment, two regression models were fitted using Matlab’s fitlm() function, one using only rotational speed as a predictor variable, and one using both forward and rotational speed. Adjusted *R*^2^ values were obtained from the output of the fitlm() function; note that all of these *R*^2^ values are adjusted for the degrees of freedom in the model fit, which allows us to compare the explanatory powers of models having different numbers of free parameters.

#### Visual learning network model

Our model was a modified version of a published model^29^ (https://research.janelia.org/jayaraman/Kim_etal_Nature2019_Downloads). In this model, the dynamics of the EPG population are given by

1 $$\begin{array}{c}\tau \frac{\text{d}{f}_{n}}{\text{d}t}=-{f}_{n}+[\alpha {f}_{n}+D({f}_{n+1}+{f}_{n-1})-\beta {\sum }_{m=0}^{N-1}{f}_{m}+1\\ \,\,-v({f}_{n+1}-{f}_{n-1})/2+{I}_{n}]{}_{+}\end{array}$$

in which [x]_+_ denotes that we are taking the non-negative values of x, *f*_*n*_ is the activity of EPG neuron *n*, *τ* is a decay time constant (50 ms), $a$ is the strength of self-excitation, *D* is the strength of excitation from neighbouring neurons, *β* is the strength of global inhibition, and *v* is a rotational velocity signal that can be positive (rightward rotation) or negative (leftward rotation). ${I}_{n}$ is the inhibitory visual input to EPG neuron *n*:

2 $${I}_{n}=-\hspace{-1mm}{\sum }_{m}{w}_{n,m}\,{g}_{m}$$

in which *w*_*n*,*m*_ is the strength of the synapse from visual neuron *m* onto EPG neuron *n*, and *g*_*m*_ is the activity of visual neuron *m*. Weights are constrained to the range [0 0.33] and visual neuron activity is constrained to the range of [0 0.35]. Note that ${I}_{n}\le 0$, as the vectors **w** and **g** contain only non-negative values. Ensemble visual neuron activity **g** was modelled as a von Mises function ($\kappa =15$) over 1D azimuthal space, with the minimal value of *g*_*m*_ set to zero. To simulate visual noise, we generate random samples from a uniform distribution over the range [0, 0.5*g*_max_] and then apply temporal smoothing (box-car averaging over 80 ms); this noisy fluctuation was then added to *I*_*n*_. The Hebbian learning rule at visual synapses onto EPG neurons was assumed to be postsynaptically gated, meaning that learning can occur only at synapses onto active EPG neurons:

3 $$\Delta {w}_{n,m}=\eta [\,{f}_{n}]{}_{+}({w}_{max}-{w}_{n,{\rm{m}}}\,)-\eta [\,{f}_{n}]{}_{+}{w}_{max}\frac{{g}_{m}}{{{g}}_{0}}$$

in which *w*_max_ = 0.33, *g*_0_ = 0.33 and *η* is the learning rate (see below for more details). The first term (*η*[*f*_*n*_]_+_ (*w*_max_ *−* *w*_*n*,*m*_)) can be interpreted as nonassociative long-term potentiation that depends on postsynaptic activity, whereas the second term (*η*[*f*_*n*_]_+_ *w*_max_*g*_*m*_/*g*_0_) can be interpreted as associative long-term depression that depends on the conjunction of presynaptic and postsynaptic activity. All elements of the model noted thus far are identical to one of the model variants in ref. ^29^.

Taking this framework as a starting point, we then modified the model of ref. ^29^ in several ways. First, as the input to the model, we used rotational velocity data that we recorded from flies walking on a spherical treadmill in a virtual environment with a single visual cue (that is, the same closed-loop visual cue that we use in the training period in Fig. 4). For each model run, we combined nine different 5-min epochs of rotational velocity data in a random order. These rotational velocity values from our data were taken as the time-varying parameter $v$; they were also used to shift the bump of activity in the visual neuron population (that is, they were used to shift the von Mises function across azimuthal space). We used a time step length of 16.1 ms for our simulation to match the sampling rate of the rotational velocity data.

Next, we scaled the synaptic learning rate $\eta$ at each time point so that it was linearly dependent on the fly’s rotational speed; this choice was motivated by our finding that the activity of ExR2 dopamine neurons is quasi-linearly dependent on the fly’s rotational speed (Fig. 1c). We refer to this as an adaptive learning rate. Note that our formulation (*η* = |0.5*v*|) is different from that of ref. ^29^ (*η* = 0.5*v*^2^).

Finally, we re-ran all of the simulations with the same behavioural data sequences as inputs, but now setting the learning rate *η* to a fixed value, obtained by taking the mean value of *η* throughout the training period in the adaptive learning models. Choosing this fixed value for *η* matched the total amount of learning across the two conditions. After the training was complete, we calculated the circular correlation between the population vector averages (PVAs) of the synaptic weights of each EPG neuron and each ER neuron at each time step, using a published method^76^. Circular correlation was computed over 3,000 s of simulation time; the mean value reported is the mean of 117 simulations (trained on shuffled blocks of behavioural data) ± 95% confidence interval (1,000 bootstrap resamples).

Equation (3) represents the postsynaptically gated learning rule that is the focus of ref. ^29^. In separate simulations, we also implemented the presynaptically gated learning rule of ref. ^29^, and we confirmed that our conclusions are the same. Specifically, adding an adaptive learning rate can have the same effect. The only difference is that, for the presynaptically gated learning rule, the overall weight structure is slightly less uniform (that is, there is a slightly lower correlation between the PVA of ER output weights and EPG input weights).

For the analysis in Extended Data Fig. 5, the final weights of 16 simulations were taken as initial weights, and 3 replicates of these 16 simulations were continued for another 3,000 s of simulation time, but with Gaussian noise added to the synaptic weight matrices at each time step. The first two replicates used the synaptic learning rules described above, whereas the third had no synaptic learning at all.

#### EPG ensemble responses to ExR2 activation or hyperpolarization

In Figs. 3 and 4, Δ*F*/*F* data for each EB wedge were calculated as described above. The PVA was calculated by converting the Δ*F*/*F* data for each wedge into a vector with a direction based on the wedge’s position in the EB, and then adding those vectors to obtain the PVA for each time point. We then computed the circular distance between the cue and the PVA position for each time point; we refer to this as the ‘offset’ between cue position and the bump position^77^. We computed bump amplitude for each time point by taking the difference between the minimum and maximum Δ*F*/*F* across all eight EB wedges.

Mutual information between the PVA and the visual cue was estimated using a published method^78^ with *k* = 3 nearest neighbours, using the Python implementation from that reference; see also ref. ^79^. For Fig. 3c, mutual information was estimated using the time points within each individual rotation of the visual cue around the fly. For Fig. 3d, the estimate used all time points in the baseline or post-ExR2 activation periods (as indicated in Fig. 3c). For Fig. 4c, mutual information was estimated for each fly using all time points within the open-loop test period.

Time points when the cue was behind the fly (at positions between 150° and 210°) were excluded from the calculations of offset and mutual information because the fly should have been unable to see the cue during these time points; including these data did not change the conclusion from either analysis.

#### Correlating bump amplitude and locomotor speed

In Fig. 4e and Extended Data Fig. 9c, speed data were collected through FicTrac 2.1 (ref. ^68^) at about 60 Hz, downsampled to the imaging data volume rate, smoothed 10 times with a Gaussian kernel 60 ms in width, and shifted back 2 imaging volumes (about 300 ms) in time to maximize correlation between the signals. Bump amplitude was calculated as maximum − minimum Δ*F*/*F* across all eight EB wedges at each time point, and then smoothed with a Gaussian kernel 600 ms in width. Then, for each fly, bump amplitude was *z*-scored and binned according to the fly’s rotational speed at each time point; and the mean bump amplitude was then calculated within each bin. Bin edges were the same for each fly and were chosen so that the number of samples in each bin was the same after aggregating data across flies. Time points with rotational speeds exceeding 100° s^−1^ (<3% of the total samples) were excluded from the analysis to ensure consistency across flies, because some individuals had higher walking speeds than others. For Fig. 4e and Extended Data Fig. 9c, the Pearson correlation coefficient between bump amplitude and rotational speed throughout the entire training and test period was calculated in Matlab using the corrcoef() function.

#### Whole-cell recording EPG neuron visual tuning

To describe the preferred cue position of an EPG neuron based on its membrane voltage, we computed its vector phase for each full cue rotation using the following equation^80^:

4 $${\rm{vector}}\,{\rm{phase}}={\rm{atan}}2({\sum }_{n}{V}_{\theta }\,\sin \theta /\,{\sum }_{n}{V}_{\theta }\,\cos \theta )$$

in which the cue position (*θ*) ranges from 0 to 360° and *V*_*θ*_ is the membrane voltage at a given stimulus angle. To adapt this analysis to the 270° azimuthal extent of the visual panorama used in our electrophysiology experiments, *θ* was obtained by rescaling and shifting cue positions from −123.75°–146.25° to 0–360°. The cell’s preferred cue position was then obtained by rescaling and shifting the calculated vector phase to the range [−123.75, +146.25°]. To determine the preferred cue position based on EPG spiking (Extended Data Fig. 6e), we calculated the vector phase as follows:

5 $${\rm{vector}}\,{\rm{phase}}={\rm{atan}}2({\sum }_{n}\sin \theta /\,{\sum }_{n}\cos \theta )$$

in which *θ* is the list of cue positions that were present at the time of a spike.

In Fig. 2d, the circular standard deviation of the preferred cue position was calculated over a pre-stimulus-window (−11 min to −1.5 min) and a post-stimulus window (3 min to 12.5 min). Changes in preferred cue position tuning for each cell were assessed using the parametric Watson–Williams multi-sample test (implemented through circ_wwtest in Matlab^77^), with Bonferroni correction. Specifically, we compared the preferred cue position values in a pre-stimulus window (−3.5 min to −1.5 min) to the preferred cue position values in a post-stimulus window (3 min to 5 min), as shown in Fig. 2c. We chose windows that are relatively narrow and closely spaced to minimize the contribution of the slow representational drift in the EPG ensemble that occurs even in control experiments over long time intervals. This drift is clearly distinct from the abrupt changes in preferred cue position that often accompany ExR2 stimulation or dopamine application, as shown in Fig. 2c. A small subset of cells were not significantly tuned to the visual cue position in the baseline period before ExR2 application or dopamine application (that is, they did not pass the Rayleigh test for uniformity with a threshold of *P* = 0.05). These cells were excluded from further statistical analysis of change in preferred cue position because the preferred cue position is not a meaningful value if there is no baseline tuning. For ExR2 activation, 11 cells were recorded, and of these, 2 cells were excluded for non-significant baseline tuning, and 6 out of the 9 remaining cells showed significant changes in preferred cue position after ExR2 activation (Bonferroni-corrected *P* = 1.9 × 10^−14^, 4.4 × 10^−14^, 1.3 × 10^−9^, 6.6 × 10^−5^, 0.0018, 0.011, 4.15, 4.51, 7.99). For the control genotype, 10 cells were recorded, 0 cells were excluded, and 0 cells showed significant changes in preferred cue position after ATP application (Bonferroni-corrected *P* = 0.064, 0.33, 0.56, 0.57, 1.95, 5.49, 6.26, 6.62, 6.99, 7.15). For dopamine application, 12 cells were recorded, and of these, 1 cell was excluded for non-significant baseline tuning, and 3 out of the remaining 11 cells showed significant changes in preferred cue position after dopamine application (Bonferroni-corrected *P* = 0.0066, 0.044, 0.045, 0.097, 1.17, 1.84, 1.86, 5.08, 5.70, 4.22, 6.89). Example cells 3, 4 and 5 that are shown in Fig. 2c showed significant changes in mean preferred cue position, whereas example cells 1, 2 and 6 did not.

Extended Data Figure 7 shows the changes in preferred cue position for every cell, including the three with non-significant baseline tuning. The three cells that failed the Rayleigh test were not excluded from any other analyses, because no other analyses would be confounded by the lack of baseline tuning in these cells.

For Fig. 2e,f and Extended Data Fig. 6a,b,f, the cell’s visual response amplitude was computed by smoothing the membrane voltage using a third-order median filter with a 100-ms window (medfilt1() Matlab function), and then subtracting the minimum voltage from the maximum voltage on each cue rotation cycle. This voltage difference was reported directly (Fig. 2b and Extended Data Fig. 6a) or normalized to the mean response during a baseline period (Fig. 2e,f and Extended Data Fig. 6f; −3.5 to −1.5 min), or reported as post − pre (Extended Data Fig. 6b; pre is −3.5 min to −1.5 min; post is 4 min to 6 min). Mean responses across cells were calculated by taking the mean of all the single-cell measurements in bins of 40 s.

For Extended Data Fig. 8, the cycle-by-cycle circular standard deviation and visual response amplitude were calculated for the full 2-min trials that preceded or followed the optogenetic stimulus in the same manner as described above. Response amplitude was calculated by taking the mean voltage for each cue position across the eight cue rotations per trial and subtracting the maximum voltage from the minimum voltage.

#### ExR2 and EPG spiking

ExR2 and EPG spikes were detected from whole-cell recordings by a custom-written Matlab script. To detect ExR2 spikes, first the raw voltage was multiplied by −1 and low-pass filtered with a digital Butterworth filter using a cutoff frequency chosen by the user. Then, the derivative was taken of this filtered signal. We detected peaks in the derivative trace that passed above a user-chosen threshold, were wider than 2.5 ms, and followed the preceding peak by more than 2 ms. Values of the cutoff frequency for the low-pass filter and the differentiated peak threshold were chosen for each cell empirically. The peristimulus time histograms of spike rate were calculated using a bin size of 5 s. Mean baseline firing rates for each cell were as follows: 0.28, 0.26, 0.04, 0.52 and 0.02 Hz. Automatic EPG spike identification (Extended Data Fig. 6c,e) was based on the detection of transients in the current output from the Axopatch 200B amplifier^81^; these transients were counted as spikes if a fluctuation wider than 1.5 ms occurred in the voltage channel within 1.5 ms of that sample, and if the peak voltage during that fluctuation was in the top 15% of values for that trial.

### Data inclusion

In one imaging experiment, an air bubble formed near the laser path soon after the beginning of the experiment (confirmed by eye with light microscopy at the end of the experiment), resulting in a marked reduction in brightness and signal-to-noise ratio, so this experiment was excluded from analysis.

For electrophysiology, cells were considered healthy and included in the analysis if their voltage was below −30 mV. For pharmacology experiments, the cell’s membrane voltage needed to remain healthy until 11 min into the treatment period to be analysed. If cells became more depolarized than −30 mV following this time point that data were excluded from analysis.

As previously reported^28^, EPG neuron electrophysiological recordings occasionally exhibit large inhibitory postsynaptic potentials with a stereotyped sharp onset, a large amplitude (>10 mV) and a stereotyped time course. They are followed by a prolonged period of depolarization when the variance of the voltage trace is also diminished. These inhibitory events interfered with visual tuning measurements, and so for Fig. 2 and Extended Data Figs. 6–8, if an event occurred it was clipped out. Such clipping was required for 12% of trials. For one cell (fly 543, cell 1), the first 12 s of the first baseline trial had too much holding current applied. Those 12 s were also excluded from the analysis.

### Statistics and sample sizes

Normality was evaluated with a Kolmogorov–Smirnov test or a Shapiro–Wilk test with *α* = 0.05. In cases when our data were not normally distributed, a nonparametric test was used. To analyse circular variables, we used statistical tests for circular statistics from the Matlab toolbox CircStat^77^. Sample sizes were chosen on the basis of standard sample sizes in the field.

### Reporting summary

Further information on research design is available in the Nature Portfolio Reporting Summary linked to this article.

## Online content

Any methods, additional references, Nature Portfolio reporting summaries, source data, extended data, supplementary information, acknowledgements, peer review information; details of author contributions and competing interests; and statements of data and code availability are available at 10.1038/s41586-022-05485-4.

### Supplementary information

Reporting Summary

## Data Availability

The hemibrain v1.2.1 connectome data are available at https://neuprint.janelia.org (also at 10.25378/janelia.11676099.v2). The datasets generated during the current study are available from the corresponding author on reasonable request.

## Code availability

Custom code is available at 10.5281/zenodo.8056588.
