# Author Correction: Dopamine promotes head direction plasticity during orienting movements

**DOI:** 10.1038/s41586-023-06238-7

**Published:** 2023-05-24

**Authors:** Yvette E. Fisher, Michael Marquis, Isabel D’Alessandro, Rachel I. Wilson

**Affiliations:** 1 Department of Neurobiology, Harvard Medical School, Boston, MA USA; 2 Present Address: Department of Molecular and Cellular Biology, University of California Berkeley, Berkeley, CA USA; 3 Present Address: Chan Zuckerberg Biohub, San Francisco, CA USA

**Keywords:** Spatial memory, Navigation

Correction to: *Nature* 10.1038/s41586-022-05485-4 Published online 30 November 2022

In the version of this article initially published, the deposit listed in the Code availability section did not include licence information for previously published code; the section is now updated to read “Custom code is available at 10.5281/zenodo.8056588” in the HTML and PDF versions of the article.

